# Skeletal abnormalities caused by a Connexin43_R239Q_ mutation in a mouse model for autosomal recessive craniometaphyseal dysplasia

**DOI:** 10.1038/s41413-024-00383-z

**Published:** 2025-01-23

**Authors:** Yasuyuki Fujii, Iichiro Okabe, Ayano Hatori, Shyam Kishor Sah, Jitendra Kanaujiya, Melanie Fisher, Rachael Norris, Mark Terasaki, Ernst J. Reichenberger, I-Ping Chen

**Affiliations:** 1 Department of Endodontology, School of Dental Medicine, University of Connecticut Health, Farmington, CT USA; 2 Department of Cell Biology, University of Connecticut Health, Farmington, CT USA; 3 Center for Regenerative Medicine and Skeletal Development, School of Dental Medicine, University of Connecticut Health, Farmington, CT USA

**Keywords:** Pathogenesis, Diseases

## Abstract

Craniometaphyseal dysplasia (CMD), a rare craniotubular disorder, occurs in an autosomal dominant (AD) or autosomal recessive (AR) form. CMD is characterized by hyperostosis of craniofacial bones and metaphyseal flaring of long bones. Many patients with CMD suffer from neurological symptoms. The pathogenesis of CMD is not fully understood. Treatment is limited to craniofacial surgery. Here, we report a knock in (KI) mouse model for AR CMD carrying a Cx43_R239Q_ mutation. *Cx43*^*KI/KI*^ mice replicate typical features of AR CMD, including thickening of craniofacial bones, club-shaped femurs, and widened diaphyseal cortical bones. Female *Cx43*^*KI/KI*^ mice display remarkably more bone overgrowth than male *Cx43*^*KI/KI*^ mice as they age. In contrast to *Cx43*^*+/+*^ littermates, *Cx43*^*KI/KI*^ mice exhibit periosteal bone deposition and increased osteoclast (OC) numbers in the endosteum of long bones. Although formation of resting OCs in *Cx43*^*+/+*^ and *Cx43*^*KI/KI*^ mice is comparable, the actively resorbing *Cx43*^*KI/KI*^ OCs have reduced resorption on bone chips. *Cx43*^*KI/KI*^ mice display reduced osteocyte dendrites. RNA from *Cx43*^*KI/KI*^ femoral cortical bones show reduced expression levels of *Sost*, *Tnf-α*, *IL-1β*, *Esr1, Esr2*, and a lower *Rankl/Opg* ratio. Moreover, the Cx43_R239Q_ mutation results in altered spatial expression of Cx43 protein and mild reduction of gap junction and hemichannel activity. The distinct phenotype seen in *Cx43*^*KI/KI*^ mice but not in Cx43 ablation models suggests that Cx43 loss-of-function is unlikely the main cause of AR CMD. Additional studies are required to investigate new roles of CMD-mutant Cx43.

## Introduction

Craniometaphyseal dysplasia (CMD) is a rare genetic bone disorder characterized by progressive hyperostosis of craniofacial bones and flared metaphyses of long bones.^1^ Diagnosis of CMD is based on clinical findings, radiographic examination, and genetic analysis. Craniofacial features of patients with CMD include hypertelorism, widened nasal bridge, paranasal bossing, widely spaced eyes with increased zygomatic width, and prominent mandibles.^2^ Progressive thickening of craniofacial bones can lead to compression of cranial nerves resulting in neurological symptoms, including visual or hearing impairment, and facial palsy.^3–5^ Associated Chiari I malformation can result in severe headaches.^6^ CMD is often diagnosed early during infancy due to difficulties in breathing and feeding. Radiographic images show hyperostotic calvarial and facial bones, sclerotic skull base, and thickened cortical bones of all proximal phalanges.^3^ CMD occurs as an autosomal dominant (AD, OMIM 605145) trait with mutations in the progressive ankylosis (*ANKH*) gene and in an autosomal recessive form (AR, OMIM 121014) carrying a R239Q mutation in Connexin 43 (CX43), encoded by the gap junction protein alpha 1 (*GJA1*) gene.^7–9^ Laboratory findings include normal or transiently decreased blood calcium and phosphate, elevated serum alkaline phosphatase (ALP), normal parathyroid hormone (PTH).^10,11^ There are no specific biomarkers for CMD. To date, CMD is managed by decompression surgery to relieve neurological symptoms. Effective pharmaceutical therapy for CMD is still missing largely because the pathogenesis of CMD remains incompletely understood.

To study the pathogenesis of CMD, we have generated an AD CMD mouse model (*Ank*^*KI/KI*^ mice) expressing an ANK_F377del_ mutation.^12^ *Ank*^*KI/KI*^ mice mimic characteristic features of CMD, including hyperostosis of craniofacial bones and flaring metaphyses of long bones. Here we generated an AR CMD mouse model (*Cx43*^*KI/KI*^) by introducing a Cx43_R239Q_ mutation. Connexins are encoded by 21 genes in humans and 20 genes in mice and are expressed in nearly all tissues.^13^ Each connexin has four transmembrane domains, two extracellular loops, one intracellular loop, and intracellular amino and carboxyl termini.^14^ Six connexins form a hemichannel that may serve as a pore or may join head to head with a hemichannel of a neighboring cell to form a gap junction to allow exchange of small molecules (<1 kD) and ions between adjacent cells.^15,16^ Cx43 is the predominant gap junction detected in osteoblasts, osteoclasts, osteocytes, and chondrocytes, although Cx45, Cx46, Cx26, and Cx37 are also expressed in bone cells.^17–23^ Mutations in connexin genes are associated with a large variety of disorders affecting many organs.^24^ Specific to bone, mutations in Cx43 are also responsible for oculodentodigital dysplasia (ODDD), characterized by syndactyly, microphthalmia, craniofacial and dental abnormalities.^9,25,26^ Cx43-related diseases suggest that other connexins cannot sufficiently compensate for mutated Cx43, which leads to predominant effects in bone cells.

The roles of Cx43 in skeletal development and bone homeostasis have been studied in multiple mouse models with Cx43 deficiencies. Global *Cx43*-null mice exhibit delayed endochondral and intramembranous ossification in addition to neural crest cell migration defects.^27^ Cx43 has been conditionally deleted in early osteochondro progenitors (*Dermo1-Cre;Cx43*^*-/fl*^ mice),^28,29^ osteoblasts (*ColI-Cre;Cx43*^*-/fl*^ mice),^30–32^ mature osteoblasts and osteocytes (*Ocn-Cre;Cx43*^*-/fl*^ mice; *Dmp1-Cre;Cx43*^*fl/fl*^ mice).^33–36^ Moreover, mice carrying different missense point mutations in *Gja1* have been generated to study ODDD.^37–39^ These *Cx43* mutant mice replicate human ODDD phenotypes including craniofacial bone anomalies, syndactyly, and enamel hypoplasia. ODDD mutations in Cx43 result in reduced formation and function of Cx43.^37–41^ Conditional Cx43 knockout mouse studies have shown that Cx43 affects mineral density, geometrical, and biomechanical properties of the skeleton as well as bone cell response to fracture and mechanotransduction by acting on osteoblasts, osteoclasts, and osteocytes, however, some findings remain controversial.^28–36^

Our group identified a novel missense mutation (c.716 G > A, p.Arg239Gln), located in the C-terminus of *GJA1*, in six patients with AR CMD by whole-exome sequencing.^9^ We introduced the Cx43_R239Q_ mutation in mice by CRISPR/Cas9 technology. *Cx43*^*KI/KI*^ mice display many features of AR CMD and do not completely phenocopy the *Ank*^*KI/KI*^ mouse model for AD CMD. Interestingly, *Cx43*^*KI/KI*^ mice also exhibit unique bone remodeling features that have not been reported in other global or conditional *Cx43* knockout mice. Bone overgrowths are observed and become more evident in aged *Cx43*^*KI/KI*^ mice. This model, together with the AD CMD model, is useful to elucidate how skeletal development, remodeling, and metabolism are altered in CMD.

## Results

### Generation and characterization of Cx43_R239Q_ KI mice, a model for AR CMD

We introduced the Cx43_R239Q_ mutation identified in AR CMD patients into the genome of C57Bl/6 J mice using CRISPR/Cas9 mediated gene editing. *Cx43*^*KI/KI*^ mice were viable and fertile. The ratio of *Cx43*^*+/+*^, *Cx43*^*+/KI*^ and *Cx43*^*KI/KI*^ litters followed Mendelian distribution (*Cx43*^*+/+*^:*Cx43*^*+/KI*^:*Cx43*^*KI/KI*^ = 64:96:58, *n* = 218). Approximately 40% of *Cx43*^*+/KI*^ breeder pairs lost some pups during nursing, likely due to a reduced feeding capability indicated by lack of milk spots in some mice (Fig. S1a). *Cx43*^*+/+*^ and *Cx43*^*KI/KI*^ mice were indistinguishable at birth and had comparable weight gain between 3 and 12 weeks of age (Fig. S1b). Male *Cx43*^*KI/KI*^ mice showed significantly increased femur length compared to *Cx43*^*+/+*^ littermates (Fig. S1c).

**Fig. 1:**
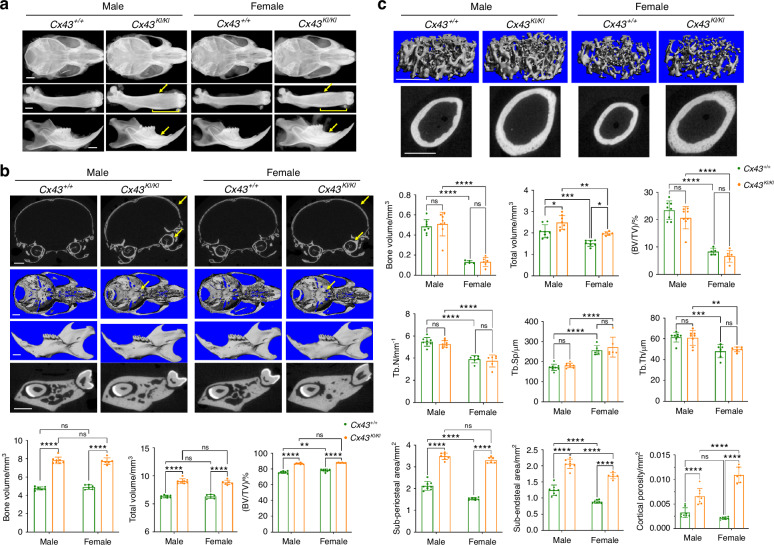
Skeletal analysis of male and female *Cx43*^*+/+*^ and *Cx43*^*KI/KI*^ mice at age of 3 months. **a** Representative radiographic images of skulls, femurs, and mandibles; Scale bar = 1 mm. Yellow arrows indicate thickened diaphyseal cortical bone and mandibular alveolar bone. Yellow brackets indicate flared metaphyses. Representative μCT images of (**b**) cross-sections of calvariae, 3D images of cranial base, mandibles, and cross-sections of mandibles through the furcation of 1st molar; yellow arrows indicate thickened calvariae and narrowed cranial foramina in *Cx43*^*KI/KI*^ mice. Scale bar = 1 mm; Histograms show quantitative measurements of BV, TV, and BV/TV. **c** Trabecular bone in metaphyses and cortical bone in the mid-diaphyses. Scale bar = 1 mm. Histograms show quantitative measurements of bone parameters by μCT analysis. Statistics were performed by two-way ANOVA followed by Tukey’s post hoc test (* *P* < 0.05, ** *P* < 0.01, *** *P* < 0.005 and **** *P* < 0.000 1). Data presented = mean ± SD

To examine whether *Cx43*^*KI/KI*^ mice replicate CMD-like skeletal phenotypes, we first performed radiographic imaging and μCT analysis in male and female *Cx43*^*+/+*^ and *Cx43*^*KI/KI*^ mice at the age of 3 months. *Cx43*^*KI/KI*^ mice had increased radiopacity of craniofacial bones, club-shaped femurs with thickened diaphyseal cortical bone, and increased alveolar bone mass with normal tooth eruption and positioning of cervical loops (Fig. 1a). μCT analysis showed that *Cx43*^*KI/KI*^ mice exhibited thickening of skull bones, narrowed neural foramina at the cranial base, and increased bone volume (BV), total volume (TV), and BV/TV in jawbones (Fig. 1b). In femurs, *Cx43*^*+/+*^ and *Cx43*^*KI/KI*^ mice did not significantly differ in metaphyseal measurements such as trabecular bone mass, trabecular number, trabecular spacing, and trabecular thickness but *Cx43*^*KI/KI*^ mice showed increased total volume due to widened metaphyses (Fig. 1c). *Cx43*^*KI/KI*^ mice had significantly increased sub-periosteal and sub-endosteal area, increased cortical diaphyseal porosity, as well as increased cortical thickness (*Cx43*^*+/+*^:*Cx43*^*KI/KI*^ male mice = 0.18±0.01 mm:0.23 ± 0.01 mm, *P* < 0.01; *Cx43*^*+/+*^:*Cx43*^*KI/KI*^ female mice = 0.15 ± 0.01 mm: 0.28 ± 0.02 mm, *P* < 0.01) (Fig. 1c). There were no significant differences in vertebral trabeculation between *Cx43*^*+/+*^ and *Cx43*^*KI/KI*^ mice (Fig. S2). Taken together, these data confirmed that both, male and female *Cx43*^*KI/KI*^ mice replicated many features of AR CMD patients, including skull and jawbone thickening, narrowed neural foramina of the cranial base, and widened metaphyses with hypersclerotic diaphyseal cortical bone in femurs.

### Effects of Cx43_R239Q_ mutation on bone turnover in *Cx43*^*KI/KI*^ mice

**Fig. 2:**
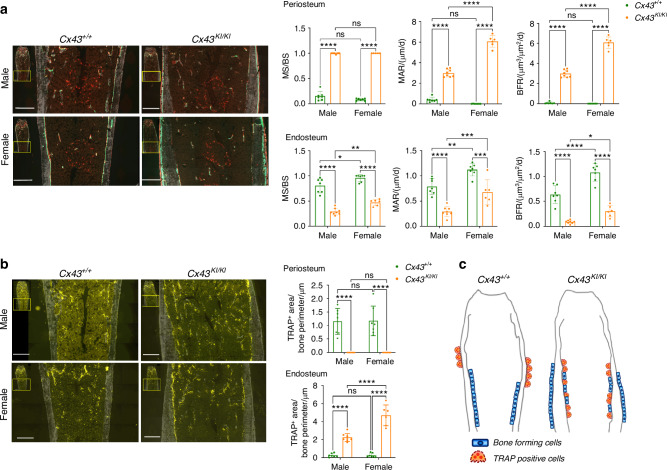
Static and dynamic histomorphometry of femoral cortical bones in male and female *Cx43*^*+/+*^ and *Cx43*^*KI/KI*^ mice at age of 3 months. **a** Representative images showing calcein (green) and alizarin complexone (red) double staining. Histograms show quantitative measurements of MS/BS, MAR, and BFR on periosteum and endosteum. **b** Representative images showing TRAP staining. Histograms show quantitative measurements of TRAP-positive stained area on periosteum and endosteum. **c** Schematic summarizing the different location patterns of bone forming cells and TRAP positive cells in *Cx43*^*+/+*^ and *Cx43*^*KI/KI*^ mice. Statistics were performed by two-way ANOVA followed by Tukey’s post hoc test. Scale bar = 500 μm. Data presented = mean ± SD (* *P* < 0.05, ** *P* < 0.01, *** *P* < 0.005 and **** *P* < 0.000 1)

To dissect out the effects of CMD-mutant Cx43 on bone turnover, we first performed static and dynamic histomorphometry in femurs of 3-month-old male and female *Cx43*^*+/+*^ and *Cx43*^*KI/KI*^ mice. Consistent with findings from μCT, the Cx43_R239Q_ mutation affects bone turnover in cortical bone more than in metaphyseal trabeculae. There were no significant differences in mineral apposition rate (MAR), bone formation rate (BFR), osteoblast surface (AP/BS) including bone forming (AP_L/BS) and bone lining surfaces (AP_NL/BS), osteoclast surface (TRAP/BS) including bone resorbing surface (TRAP_L/BS) and bone remodeling surface (TRAP_NL/BS) in femoral metaphyses of *Cx43*^*+/+*^ and *Cx43*^*KI/KI*^ mice (Table 1). In general, female mice exhibited increased osteoclast surface (TRAP/BS) and bone remodeling unit surface (AP_TRAP_R/BS) than male mice (Table 1). On the other hand, diaphyseal cortical bones of *Cx43*^*+/+*^ and *Cx43*^*KI/KI*^ mice showed distinctly different patterns. Active bone formation, determined by the presence of calcein (green) and alizarin complexone (red) labeling, was observed on the periosteal surface of *Cx43*^*KI/KI*^ mice but mostly on the endosteal surface of *Cx43*^*+/+*^ mice (Fig. 2a). The mineralizing surface/bone surface (MS/BS), mineral apposition rate (MAR), and bone formation rate (BFR) were significantly increased in the periosteum of *Cx43*^*KI/KI*^ mice compared to *Cx43*^*+/+*^ mice (Fig. 2a). In contrast, MS/BS, MAR, and BFR in the endosteum were decreased in *Cx43*^*KI/KI*^ mice (Fig. 2a). We next analyzed TRAP-positive cells, indicative of pre-osteoclastic cells and osteoclasts (OCs), in cortical bones of the mid-diaphyseal region of femurs. While *Cx43*^*+/+*^ mice showed some TRAP staining on the periosteal surface, mostly below the widest portion of metaphyses, *Cx43*^*KI/KI*^ exhibited significantly increased TRAP-positive cells on the endosteal surface and very little on periosteum (Fig. 2b). Our data, summarized in Fig. 2c, show an opposite location pattern of bone forming cells and TRAP-positive cells in *Cx43*^*+/+*^ and *Cx43*^*KI/KI*^ mice, which may help to explain the substantial cortical bone thickening in the diaphysis and an enlarged bone marrow cavity with club-shaped femurs in *Cx43*^*KI/KI*^ mice. Interestingly, differences between *Cx43*^*+/+*^ and *Cx43*^*KI/KI*^ mice in the sub-periosteal area, cortical porosity, MAR (periosteum), BFR (periosteum), BFR (endosteum), and TRAP^+^ area/bone perimeter (endosteum) were more remarkable in female than in male mice (Table S1).

**Table 1:** Dynamic and static histomorphometry of trabecular parameters in metaphyses of femurs of 13-week-old *Cx43*^+/+^ and *Cx43*^*KI/KI*^ male and female mice

Parameters	Male		Female	
	*Cx43*^+/+^ (*n* = 8)	*Cx43*^*KI/KI*^ (*n* = 8)	*Cx43*^+/+^ (*n* = 8)	*Cx43*^*KI/KI*^ (*n* = 8)
MAR/ (μm/d)	1.31 ± 0.05	1.48 ± 0.11	1.37 ± 0.23	1.26 ± 0.27
BFR /(μm^3^/μm^2^/d)	0.49 ± 0.03	0.56 ± 0.07	0.47 ± 0.08	0.42 ± 0.12
(AP/BS)/%	77.94 ± 2.91	76.65 ± 7.35	80.9 ± 7.12	85.86 ± 2.29
(AP_L/BS)/%	53.83 ± 1.12	50.64 ± 7.30	51.42 ± 4.16	53.16 ± 5.18
(AP_NL/BS)/%	24.73 ± 2.04	24.96 ± 3.25	29.58 ± 3.34	32.84 ± 4.57
(TRAP/BS)/%	18.27 ± 5.11	13.41 ± 2.17	25.73 ± 2.92	29.74 ± 4.85
(TRAP_L/BS)/%	11.05 ± 2.62	8.72 ± 1.74	16.96 ± 2.52	18.08 ± 2.09
(TRAP_NL/BS)/%	5.48 ± 1.47	4.83 ± 0.71	10.34 ± 1.83	12.30 ± 3.58
(AP_TRAP_R/BS)/%	8.31 ± 2.22	6.13 ± 1.77	11.58 ± 1.97	13.13 ± 1.55

R: alizarin complexone labeling. Data presented: mean ± SD. No significant differences between sex matched *Cx43*^*+/+*^ and *Cx43*^*KI/KI*^ mice were noted by two-way ANOVA with Tukey’s multiple comparison test

*MAR* mineral apposition rate, *BFR* bone formation rate, *AP* alkaline phosphatase staining, *BS* bone surface, *AP/BS* AP^+^ surface per bone surface, *L* labeling surface, *AP_L/BS* AP^+^ over labeling surface per bone surface, *NL* non-labeling surface, *AP_NL /BS* AP^+^ over non-labeling surface per bone surface, *TRAP* tartrate-resistant acid phosphatase, *TRAP/BS* fraction of bone surface with TRAP label, *TRAP_L/BS* proportion of mineralizing surface that is covered with the TRAP label, *TRAP_NL/BS* proportion of non-mineralizing surface that is covered with the TRAP label, *AP_TRAP_R/BS* proportion of bone surface where the AP, TRAP and mineralization signals are co-localized.

**Fig. 3:**
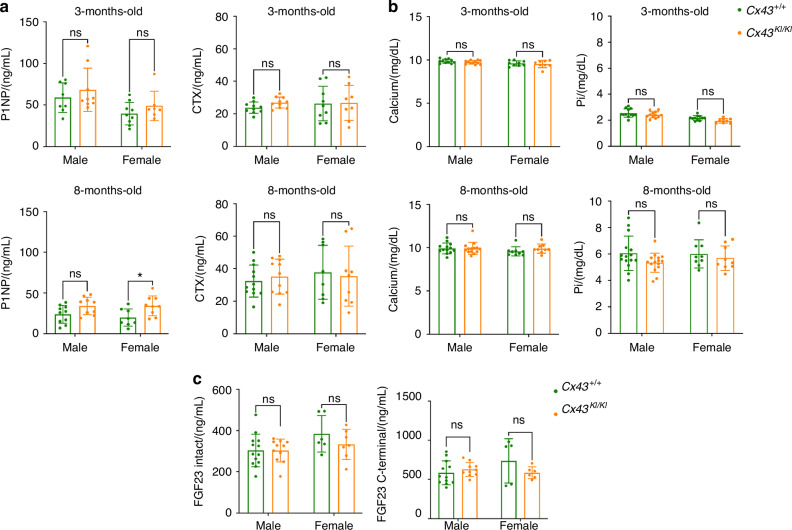
Biochemical analysis of male and female *Cx43*^*+/+*^ and *Cx43*^*KI/KI*^ mice at ages of 3 and 8 months. **a** Serum levels of P1NP, a marker for bone formation, and CTX, a marker for bone resorption. **b** Serum levels for Ca and Pi. **c** Serum levels for the intact (active) and C-terminal (inactive) form of FGF23 in 8-month-old mice. Statistics was performed by two-way ANOVA followed by Tukey’s post hoc test for comparison between *Cx43*^*+/+*^ and *Cx43*^*KI/KI*^ mice (* *P* < 0.05)

We next measured serum levels of P1NP, a marker for bone formation, and CTX, a marker for bone resorption, in male and female *Cx43*^*+/+*^ and *Cx43*^*KI/KI*^ mice at ages of 3 and 8 months. Serum levels of P1NP were similar in 3-month-old *Cx43*^*+/+*^ and *Cx43*^*KI/KI*^ mice but were significantly increased in 8-month-old female *Cx43*^*KI/KI*^ mice when compared to female *Cx43*^*+/+*^ mice of the same age (Fig. 3a). Serum levels of CTX remained comparable between *Cx43*^*+/+*^ and *Cx43*^*KI/KI*^ mice at ages of 3 and 8 months (Fig. 3a). 8-month-old mice showed remarkable reduction of P1NP levels and elevation of CTX levels compared to 3-month-old mice, suggesting somewhat decreased bone formation and increased bone resorption with aging.

Serum calcium (Ca) and phosphate (Pi) levels in patients with AR CMD were reported to be within normal range or slightly decreased.^9,42^ In *Cx43*^*+/+*^ and *Cx43*^*KI/KI*^ mice, serum Ca and Pi levels were comparable, both at ages of 3 and 8 months (Fig. 3b). We then measured fibroblast growth factor 23 (FGF23) levels, a phosphaturic factor secreted by bone to promote Pi wasting in kidney that is associated with several bone disorders.^43,44^ Intact FGF23 protein levels in the *Ank*^*KI/KI*^ model for AD CMD, and in AD CMD patients were within normal range.^10,45^ Our data showed that there were no significant differences in intact FGF23 and C-terminal FGF23 protein levels between *Cx43*^*+/+*^ and *Cx43*^*KI/KI*^ mice (Fig. 3c).

### Progressive worsening of bone phenotype as *Cx43*^*KI/KI*^ mice age

We next analyzed skeletal phenotypes in aging mice since CMD progresses throughout life. Male and female *Cx43*^*KI/KI*^ mice progressively developed bone overgrowths in multiple sites of craniofacial and long bones, including but not limited to femurs and mandibles, shown by radiographs taken at ages of 4, 5, 6, and 7 months (Fig. S3a). Consistent with radiographs, μCT analysis for bones of 7-month-old mice showed grossly increased bone overgrowths in mandibles and femurs (Fig. S3b).

**Fig. 4:**
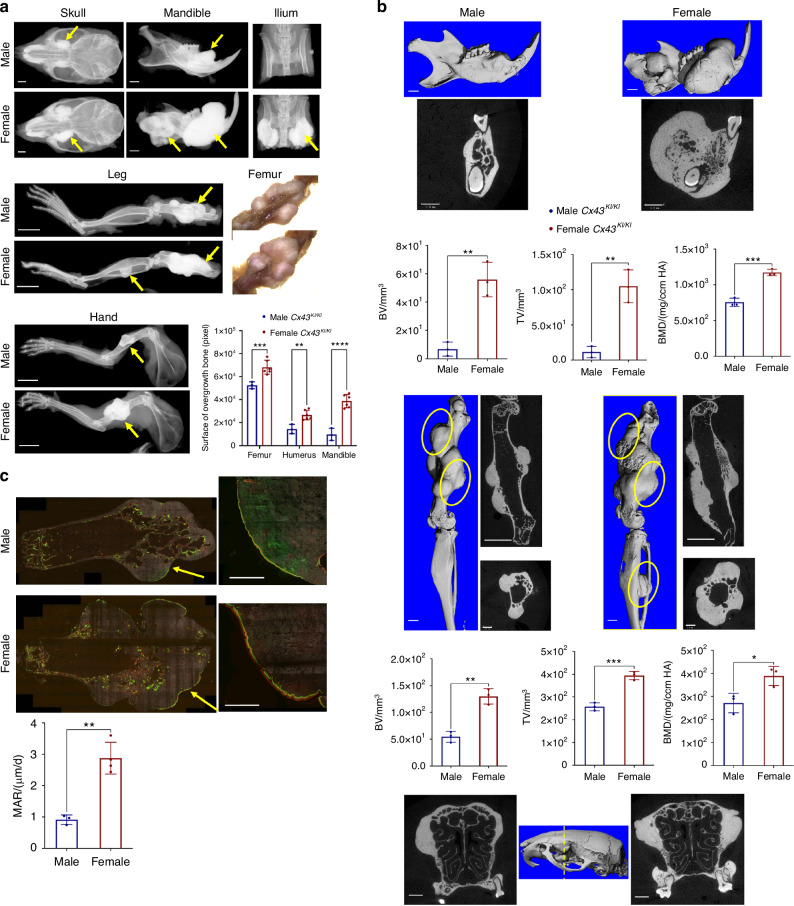
Skeletal analysis of 1-year-old male and female *Cx43*^*KI/KI*^ mice. **a** Representative radiographs of skulls, mandibles, iliac bones, legs and hands; femoral bone, scale bar = 1 mm, leg and hand, scale bar = 5 mm. **b** Representative 3D μCT images of mandibles and femurs, 2D images of cross-sections of mandibles, femurs (longitudinal and cross sections), and skulls (cross section through the yellow dotted line); yellow circles indicate bone overgrowth, scale bar = 1 mm. *n* = 3/group. **c** Dynamic histomorphometry of femurs of male and female *Cx43*^*KI/KI*^ mice; scale bar = 500 μm. Right panels show the magnified images of the areas indicated by yellow arrows on the left panels. Histograms show statistical analysis of quantitated data by Student’s *t* test. Data presented = mean ± SD (* *P* < 0.05, ** *P* < 0.01, *** *P* < 0.005 and **** *P* < 0.000 1)

While both sexes exhibited skeletal abnormalities, female *Cx43*^*KI/KI*^ mice presented with a more prominent phenotype as they aged. At one-year-old, female *Cx43*^*KI/KI*^ mice had significantly more prominent bone overgrowths in skulls, mandibles, iliac bones, femurs, and humeri compared to male *Cx43*^*KI/KI*^ mice (Fig. 4a). μCT analysis showed significantly increased BV, TV, and bone mineral density (BMD) in mandibles and femurs of female *Cx43*^*KI/KI*^ mice (Fig. 4b). The bony overgrowths in skulls and maxillae are visibly increased in female *Cx43*^*KI/KI*^ mice (Fig. 4b). Interestingly, female *Cx43*^*KI/KI*^ mice exhibited increased MAR in the periosteum in areas of bone overgrowths, suggesting that more new bone deposition occurs in female *Cx43*^*KI/KI*^ mice (Fig. 4c).

### Effect of Cx43_R239Q_ on osteoblast and osteoclast cultures

**Fig. 5:**
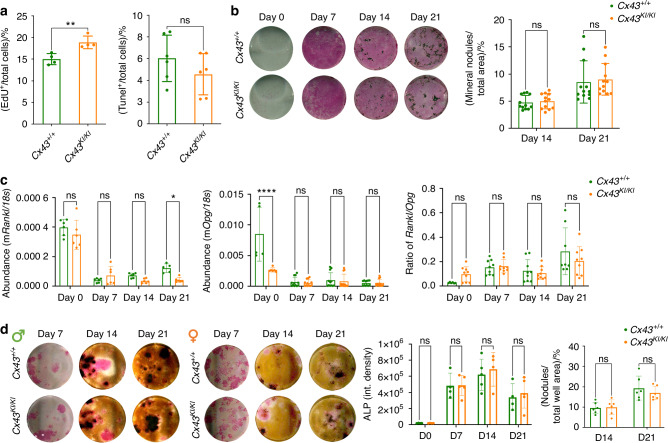
Osteoblast cultures of *Cx43*^*+/+*^ and *Cx43*^*KI/KI*^ mice. **a** Cell proliferation and apoptosis in mCOB cultures analyzed by EdU and Tunel assays, respectively. **b** Alkaline phosphatase (ALP) and mineral nodule formation by von Kossa staining. **c** *Rankl* and *Opg* mRNA expression levels in mCOB cultures. **d** ALP and mineral nodule formation in bone marrow stromal cultures (BMSCs). Histograms show no significant differences between male *Cx43*^*+/+*^ and *Cx43*^*KI/KI*^ mice. Statistical analysis was performed by two-way ANOVA followed by Tukey’s post hoc test. Data presented = mean ± SD (* *P* < 0.05, ** *P* < 0.01, **** *P* < 0.000 1)

To examine mutational effects of Cx43_R239Q_ on osteoblasts, we isolated calvarial osteoblasts (mCOBs) and bone marrow stromal cells (BMSCs) from *Cx43*^*+/+*^ and *Cx43*^*KI/KI*^ mice and cultured cells in osteogenic differentiation medium for 21 days. We examined cell proliferation and apoptosis 2 days after plating mCOBs by EdU and Tunel assays, respectively. *Cx43*^*KI/KI*^ mCOBs displayed significantly increased proliferation but comparable apoptosis in comparison to *Cx43*^*+/+*^ mCOBs (Fig. 5a). Mineral nodule formation of mCOBs cultured in osteogenic medium for 14 and 21 days were comparable between *Cx43*^*+/+*^ and *Cx43*^*KI/KI*^ mice (Fig. 5b). Expression levels of osteoblast marker genes examined by qPCR including *ColI*, *Alp*, *Opn*, *Osx*, *Runx2*, *Ocn* were comparable between *Cx43*^*+/+*^ and *Cx43*^*KI/KI*^ mCOBs (Fig. S4a). However, *Opg* at day 0 was increased and *Rankl* at day 21 was decreased in *Cx43*^*KI/KI*^ mCOBs, although the *Rankl/Opg* ratio was not significantly different for any time points (Fig. 5c). Similarly, ALP staining and mineral nodule formation were not significantly different in *Cx43*^*+/+*^ and *Cx43*^*KI/KI*^ BMSCs from male and female mice (Fig. 5d).

**Fig. 6:**
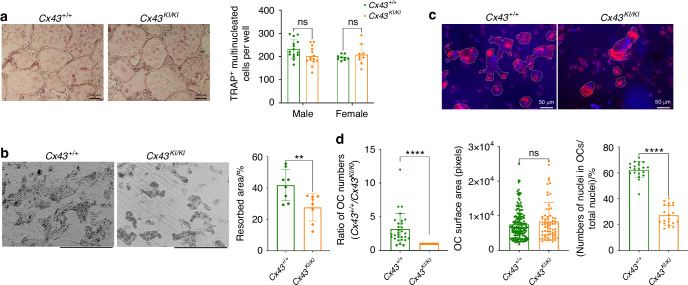
Cx43_R239Q_ mutational effects on osteoclasts (OCs). **a** Formation of resting OCs (rOCs) was comparable between *Cx43*^*+/+*^ and *Cx43*^*KI/KI*^ BMM cultures; scale bar = 200 μm. Statistical analysis was performed by two-way ANOVA followed by Tukey’s post hoc test. **b** Resorption on bone chips by actively resorbing *Cx43*^*KI/KI*^ OCs (aOCs) was reduced; scale bar = 300 μm. **c** *Cx43*^*+/+*^ and *Cx43*^*KI/KI*^ aOCs stained by rhodamine phalloidin; scale bar = 50 μm. **d** Quantitative measurements of aOCs that formed, surface area of aOCs, and aOC fusion, which is measured by nuclei numbers in aOCs divided by total nuclei numbers on bone chips. Statistical analysis was performed by Student’s *t* test (** *P* < 0.01, **** *P* < 0.000 1)

To examine the mutational effects of Cx43_R239Q_ on OCs, we first cultured bone marrow-derived macrophages (BMMs) on culture plates and induced the formation of resting osteoclasts (rOCs) by supplementation with M-CSF and RANKL. TRAP^+^ cells with more than 3 nuclei were counted as OCs. There was no significant difference in the formation of rOCs between *Cx43*^*+/+*^ and *Cx43*^*KI/KI*^ cultures (Fig. 6a). Expression levels of OC marker genes that included *Nfatc1*, *Rank*, *Cathepsin K*, *αv integrin, β3 integrin*, and *Mmp9* were comparable between *Cx43*^*+/+*^ and *Cx43*^*KI/KI*^ rOCs (Fig. S4b). We next plated BMMs on bone chips to examine the formation and function of actively resorbing osteoclasts (aOCs). Interestingly, the bone resorption pit assay showed a significant reduction of the resorptive activity of *Cx43*^*KI/KI*^ aOCs when compared to *Cx43*^*+/+*^ aOCs (Fig. 6b). We next examined the aOCs by rhodamine phalloidin staining and found that reduced numbers of aOCs formed on bone chips (Fig. 6c, d). The size of *Cx43*^*+/+*^ and *Cx43*^*KI/KI*^ aOCs were not significantly different but there was decreased fusion activity in *Cx43*^*KI/KI*^ aOCs, suggested by reduced numbers of nuclei in OCs normalized to total nuclei numbers (Fig. 6d).

### Effect of Cx43_R239Q_ on osteocytes

**Fig. 7:**
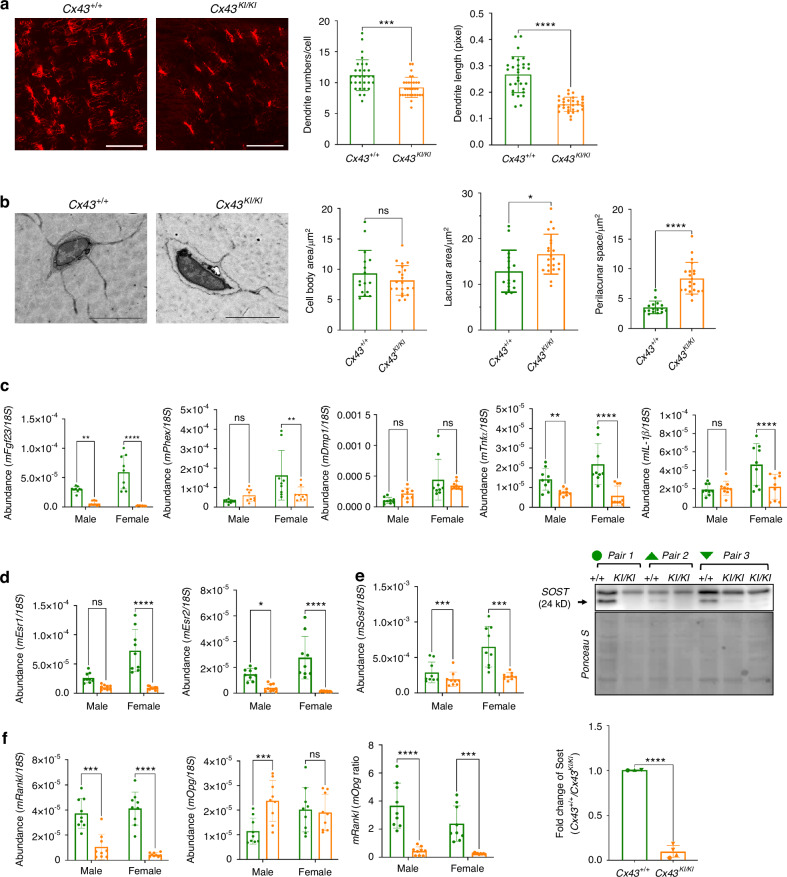
Osteocyte phenotype in *Cx43*^*KI/KI*^ mice. **a** Dendrite formation of osteocytes stained by rhodamine phalloidin; scale bar = 50 μm. **b** Osteocytes imaged by scanning electron microscopy (SEM) and quantitative measurements of areas of osteocyte bodies, lacunar area, and perilacunar space; scale bar = 5 μm. Statistical analysis in (**a**, **b**) was performed by Student’s *t* test (ns: no significant difference, * *P* < 0.05, **** *P* < 0.000 1). **c** Expression levels of *Fgf23*, *Phex*, *Dmp1*, *Tnfα*, and *IL-1β* by qPCR. **d** mRNA levels of *Esr1* and *Esr2*. **e** *Sost* mRNA and prot**e**in levels. Histogram shows fold difference of SOST levels in three pairs of *Cx43*^*+/+*^ and *Cx43*^*KI/KI*^ littermates quantified by normalizing SOST levels with Ponceau S staining. Statistical analysis was performed by Student’s *t* test (**** *P* < 0.001). **f** *Rankl* and *Opg* mRNA levels. The ratio of *Rankl/Opg* is decreased in *Cx43*^*KI/KI*^ mice. Statistical analysis was performed by two-way ANOVA followed by Tukey’s post hoc test (* *P* < 0.05, ** *P* < 0.01, *** *P* < 0.005 and **** *P* < 0.000 1)

Osteocytes are the most abundant cell type in bone and regulate the quality of bone matrix by directing osteoblasts and osteoclasts via dendrites and secreted molecules.^46^ To examine the effects of Cx43_R239Q_ on osteocytes, we examined osteocytes in diaphyseal cortical bone. Rhodamine phalloidin staining showed that dendrites of *Cx43*^*KI/KI*^ osteocytes were reduced in number and length (Fig. 7a). We next visualized osteocytes by scanning electron microscopy. The lacunar area of *Cx43*^*KI/KI*^ osteocytes was significantly increased due to increased perilacunar space but the cell body area of *Cx43*^*+/+*^ and *Cx43*^*KI/KI*^ osteocytes was comparable (Fig. 7b). Expression levels of *Fgf23* and *Phex* mRNAs in the cortex of femurs were significantly decreased in *Cx43*^*KI/KI*^ bones while *Dmp1* was comparable to *Cx43*^*+/+*^ bones (Fig. 7c). Osteocytes express pro-inflammatory cytokines such as *Tnf-α*, and *IL-1β*, which can lead to increased osteoclastogenesis and inhibit osteoblast formation.^47–49^ *Tnf-α* and *IL-1β* were also significantly reduced in *Cx43*^*KI/KI*^ bones (Fig. 7c). Female *Cx43*^*KI/KI*^ mice displayed stronger localized bone overgrowth than male *Cx43*^*KI/KI*^ mice. Therefore, we examined expression levels of *Esr1* and *Esr2* encoding estrogen receptor α and β (ERα and ERβ) *Esr2* was significantly decreased in both, male and female *Cx43*^*KI/KI*^ bones while *Esr1* was only negatively affected in female *Cx43*^*KI/KI*^ bones (Fig. 7d). Osteocytes express *Sost* to negatively regulate bone formation and *Rankl* to stimulate osteoclast activity.^50,51^ *Sost* mRNA and protein levels were significantly decreased and the *Rankl/Opg* ratio was reduced in *Cx43*^*KI/KI*^ bones (Fig. 7e, f). These data together suggest that the Cx43_R239Q_ mutation impacts the osteocyte function, at least partially, via altered osteocyte dendrite organization and expression of several key molecules that regulate bone homeostasis.

### Expression and function of Cx43_R239Q_ mutant protein

**Fig. 8:**
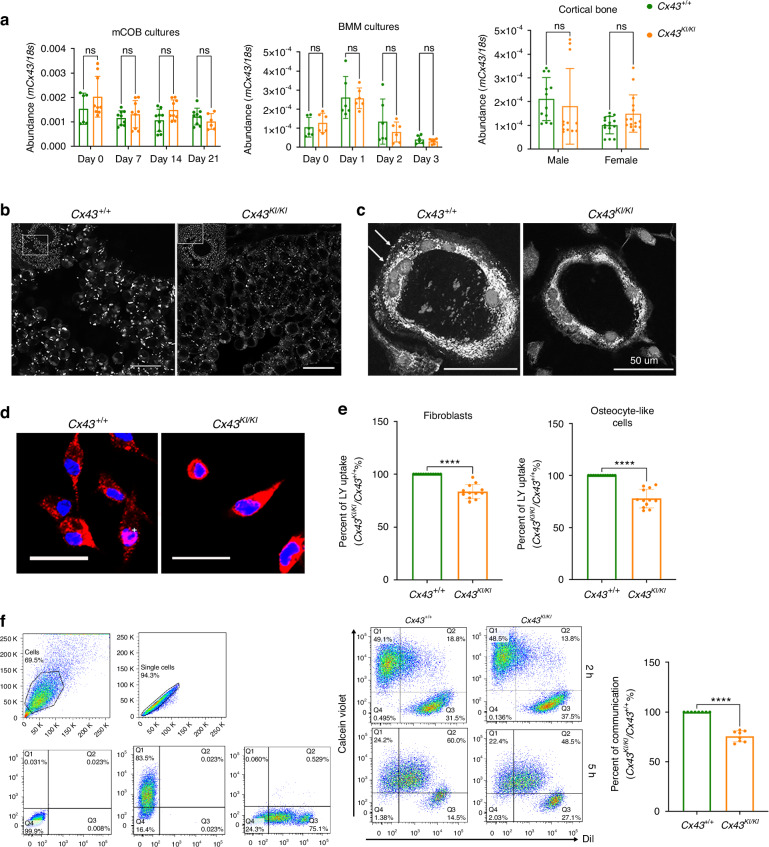
Expression, localization, and function of mutant *Cx43*^*KI/KI*^ protein. **a** Abundance of wild type and mutant *Cx43* mRNA in mCOBs, BMMs, and long bones by qPCR. **b** Localization of wild type and mutant Cx43 in ovaries; scale bar = 50 μm. **c** Localization of wild type and mutant Cx43 in OCs; scale bar = 50 μm; white arrows indicate Cx43 staining on the periphery of *Cx43*^*+/+*^ OCs. **d** Localization of wild type and mutant Cx43 in osteocyte-like cells; scale bar = 20 μm. Red: Cx43; blue: Hoechst 33342 nuclei staining. **e** Luciferase yellow dye uptake assays in fibroblasts (left panel) and osteocyte-like cells (right panel); statistical analysis performed by Student’s *t* test (**** *P* < 0.000 1). **f** Gap junction activity by flow cytometry. Forward and side scatter has been applied to select the single-cell population and to remove debris (left top panel). The gating strategies are defined by parameters obtained from cells with no staining and single staining (Calcein violet or Dil) (left bottom panel). Histogram shows the percentage of communication = [Q2/(Q2 + Q3)] x100. Q2 is the dual positive population and Q3 is the acceptor cell population stained with Dil. Statistical analysis was performed by Student’s *t* test (ns: no significant difference, **** *P* < 0.000 1)

Expression levels of *Cx43* mRNA during osteoblast and osteoclast differentiation were comparable between *Cx43*^*+/+*^ and *Cx43*^*KI/KI*^ mCOB and BMM cultures, respectively (Fig. 8a). In addition, wt and CMD mutant *Cx43* levels were not significantly different in RNA samples isolated from femoral cortical bones (Fig. 8a). We found that both, Cx43 and Cx43_R239Q_ proteins, are most abundantly expressed in brain, heart, testis/ovary and to a lesser amount in skin, bone, and liver but were undetectable in kidney (Fig. S5a). Comparable levels of Cx43 and Cx43_R239Q_ protein were also shown in lysate collected from mCOBs, BMMs, and osteocyte-enriched cultures by Cx43 immunoblots (Fig. S5b-S5d). We next examined the localization of wt and mutant Cx43 protein by immunocytochemistry. Cx43 immunostaining of ovaries showed distinct large punctae in *Cx43*^*+/+*^ mice compared to a weaker staining and more diffuse punctation in *Cx43*^*KI/KI*^ mice (Fig. 8b). Similarly, the immunocytochemistry data of *Cx43*^*+/+*^ and *Cx43*^*KI/KI*^ OCs and osteocyte-like cells showed altered spatial expression of mutant Cx43, which appeared to be more localized around the nucleus, while wt Cx43 appeared to spread out more throughout the cells (Figs. 8c, 8d, Fig. S6). Cx43 serves as a hemichannel and gap junction protein when located on the plasma membrane. To examine whether the hemichannel function is compromised, skin fibroblasts and osteocyte-like cells isolated from *Cx43*^*+/+*^ and *Cx43*^*KI/KI*^ mice were loaded with Lucifer Yellow dye and the amount of dye uptake was measured in a microplate reader. Dye uptake was, in average, reduced by 17% and 23% in *Cx43*^*KI/KI*^ fibroblasts and osteocytes, respectively, compared to *Cx43*^*+/+*^ cells (Fig. 8e). Furthermore, the gap junction activity measured by a parachute assay with DilC_18_ dye (recipient cells) and calcein violet AM dye (donor cells) also showed approximately 25% reduced cell communication in *Cx43*^*KI/KI*^ cells (Fig. 8f). In summary, the Cx43_R239Q_ mutation does not affect the expression of mutant Cx43 but results in altered spatial expression. Moreover, hemichannel and gap junction functions are mildly decreased but reach statistical significance in *Cx43*^*KI/KI*^ cells.

## Discussion

Prior to the identification of the Cx43_R239Q_ mutation for AR CMD,^9^ there were a small number of presumed AR CMD cases reported without genetic validation.^52–55^ A case report following a patient with AR CMD for 17 years showed flared metaphyses at the age of 4½ months, sclerotic change of diaphyses at age of 2 years, followed by mild expansion of metaphyses with localized new bone nodule on the shafts of fibulae at 17 years old.^54^ Another case report of a 46-year-old AR CMD patient described radiographic evidence for widened cortical diaphyses of upper long bones.^55^ *Cx43*^*KI/KI*^ mice replicate many skeletal features of AR CMD including hyperostotic craniofacial bones and widened metaphyses with hypersclerotic cortical bones. The metaphyseal flaring is more significant at younger age and, as *Cx43*^*KI/KI*^ mice age, their diaphyses widen with localized bone nodule formation. This skeletal change is consistent with reports of patients with AR CMD.^54,55^

Before genetic testing became available, some case reports of patients with severe CMD were classified as autosomal recessive without genetic evidence.^54,56^ However, patients with AR CMD develop more hypersclerotic diaphyseal cortical bones but are less severely impacted by neuronal compression in cranial bones than patients with AD CMD.^53^ Most AR CMD patients have normal to mild vision or hearing impairment while most AD CMD patients have cranial nerve compression with some degree of facial paralysis, blindness, deafness, or severe headache. We previously reported a mouse model for AD CMD (*Ank*^*KI/KI*^ mice) carrying an *ANK*_*F377del*_ mutation.^12^ When comparing *Cx43*^*KI/KI*^ mice to *Ank*^*KI/KI*^ mice, we noted that *Cx43*^*KI/KI*^ mice, even at 1 year old, have less severe narrowing of cranial foramina and do not have any joint stiffness phenotype compared to 3-month-old AD CMD *Ank*^*KI/KI*^ mice (Fig. S7). The joint stiffness phenotype of *Ank*^*KI/KI*^ mice becomes evident when *Ank*^*KI/KI*^ mice become incapable of grabbing cage bars or bending upper and lower extremities, as they develop excessive deposition of hydroxyapatite around joint spaces.^12^ Other differences between *Ank*^*KI/KI*^ mice and *Cx43*^*KI/KI*^ mice include: (1) *Cx43*^*KI/KI*^ mice are viable whereas *Ank*^*KI/KI*^ mice die around 5–6 months old; (2) *Cx43*^*KI/KI*^ mice are fertile whereas *Ank*^*KI/KI*^ mice are infertile; (3) *Cx43*^*KI/KI*^ mice develop more hypersclerotic diaphyseal cortical bone than *Ank*^*KI/KI*^ mice; (4) only *Cx43*^*KI/KI*^ mice progressively develop irregular bone overgrowths at multiple skeletal sites, a feature seen in patients with AR CMD but not in patients with AD CMD or in *Ank*^*KI/KI*^ mice.^54^

We observed several similarities and differences between our *Cx43*^*KI/KI*^ mice and other Cx43 mouse models. *Cx43*^*KI/KI*^ mice have a normal life span while the global Cx43 deletion (*Cx43*^*KO/KO*^) mice die within one hour of delivery due to heart anomalies and muscular contractions.^27^ Mice with *Cx43* ablation in early osteochondro progenitors (*DM1Cre;Cx43*^*-/fl*^), osteoblasts (*ColICre; Cx43*^*-/fl*^), mature osteoblasts/osteocytes (*OcnCre;Cx43*^*-/fl*^), and osteocytes (*Dmp1Cre;Cx43*^*fl/fl*^) and our *Cx43*^*KI/KI*^ mice display a stronger phenotype in cortical bones than in cancellous bones, which may be explained by relatively low levels of *Gja1* expression in trabecular bone. Osteocyte apoptosis caused by lack of Cx43 in mature osteoblasts/osteocytes is more pronounced in cortical bones.^28–36^ While *DM1Cre;Cx43*^*-/fl*^ and *ColICre; Cx43*^*-/fl*^ mice are osteopenic and *OcnCre;Cx43*^*-/fl*^ mice have no bone mass abnormalities,^28,30,33^ our *Cx43*^*KI/KI*^ mice display a site-specific bone phenotype, including increased bone mass in craniofacial bones and diaphyseal cortical bones but no changes in vertebrae nor metaphyseal trabecular bones. Conditional Cx43 deletion mice have a thin cortex^28,31,36^ whereas our *Cx43*^*KI/KI*^ mice exhibit cortical bone thickening. In addition, the bone overgrowths are only presented in aged *Cx43*^*KI/KI*^ mice. The shared phenotypes between *Cx43*^*KI/KI*^ and *Cx43* ablation mouse models and distinct bone abnormalities in *Cx43*^*KI/KI*^ mice suggest that AR CMD is, partially, but not merely caused by Cx43 loss-of-function. The mild reductions of dye uptake and gap junction activity may not be the main driver for the unique bone overgrowths in *Cx43*^*KI/KI*^ mice.

The AD CMD mutation in *Ank*^*KI/KI*^ mice and AR CMD mutation in *Cx43*^*KI/KI*^ mice cause some level of functional loss albeit via different mechanisms. The F377del mutation in ANK/ANKH leads to reduced levels of functional ANK/ANKH protein due to rapid protein degradation.^57^ The R239Q mutation in Cx43, on the other hand, does not affect Cx43 expression levels but causes altered spatial expression and compromised function of mutant Cx43. It remains unknown whether the decreased Cx43 gap junction/hemichannel function is due to decreased presence of mutant Cx43 on the plasma membrane or a change in conductance activity. In mouse models for ODDD that carry I130T or G318R mutations in Cx43, the abundance of Cx43 protein is decreased, the trafficking is interfered, and the Cx43-mediated conduction is impaired.^38,39^ ODDD mice and our CMD *Cx43*^*KI/KI*^ mice have distinct phenotypes. We suspect that Cx43_R239Q_ protein may enhance or inhibit activities of other molecules by altering binding specificity or affinity with other protein partners. The R239Q mutation is located at the Cx43 intracellular C-terminal domain, which may interact with various protein partners, including molecules involved in adherens junctions and with cytoskeletal proteins. More specifically, the R239Q mutation lies within the tubulin binding motif (residues 228-260).^58–60^ Interaction between Cx43 and tubulin/microtubules can affect the intracellular trafficking of Cx43, regulate gap junction function, and control the TGF-β pathway via competing with Smad2 for tubulin/microtubule binding.^59^ We will focus on investigating a yet unknown function of mutant Cx43 in AR CMD in future studies.

Some in vitro and in vivo data in this study may appear inconsistent. In vitro data represent the behavior of an enriched cell population while in vivo data show the cellular phenotype resulting from cell-autonomous and non-cell autonomous effects in specific compartments. For example, increased numbers of TRAP^+^ cells on the endosteum in *Cx43*^*KI/KI*^ mice are results of cell-autonomous effects of OCs, the crosstalk between OC precursors and neighboring cells as well as the effects of endocrine/paracrine factors in the bone marrow environment. Increased number of TRAP^+^ cells in the endosteum of *Cx43*^*KI/KI*^ mice may compensate the osteoclast function deficiencies observed in *Cx43*^*KI/KI*^ BMM cultures on bone chips. In vitro OB data are consistent with metaphyseal histomorphometry data that show no significant difference between *Cx43*^*+/+*^ and *Cx43*^*KI/KI*^ mice but obviously do not reflect the differences seen in diaphyseal cortical bones in vivo. Serum levels of CTX (bone resorption marker) and P1NP (bone formation marker) measure the overall activity but do not reflect site-specific skeletal phenotypes shown by histomorphometry in *Cx43*^*KI/KI*^ mice. For example: the number of TRAP^+^ cells is increased in the endosteum of long bones in *Cx43*^*KI/KI*^ mice but decreased in the periosteum while bone formation activity is most prominent in the periosteum but barely detectable in the endosteum.

*Cx43*^*KI/KI*^ mice have reduced sclerostin (*Sost*), which inhibits bone formation, decreased receptor activator of nuclear factor-κB ligand (*Rankl*), which promotes bone resorption, and low levels of the proinflammatory cytokines tumor necrosis factor alpha (*Tnf-α*) and interleukin 1β (*IL-1β*), which can stimulate osteoclast formation.^51,61,62^ These data taken together are in favor of increased bone mass, however, do not directly point to mechanisms for different localization of bone cells in periosteum (increased OB, reduced OC) vs. endosteum (reduced OB, increased OB) in *Cx43*^*KI/KI*^ mice. Furthermore, our data show that *Esr1* is only reduced in female *Cx43*^*KI/KI*^ mice while *Esr2* is reduced in both, male and female *Cx43*^*KI/KI*^ mice. *Esr1* and *Esr2* regulate bone mass differently in male and female mice.^63–65^ Bone remodeling in male mice is regulated only by *Esr1*, whereas bone turnover in female mice is controlled by *Esr1* and *Esr2*.^66,67^ Differentially affected levels of *Esr1* and *Esr2* between male and female *Cx43*^*KI/KI*^ mice may provide a direction for future studies to investigate the cause for more severe bone overgrowths in female *Cx43*^*KI/KI*^ mice. Lastly, we observed a discordance between the decreased mRNA level of *Fgf23* and normal levels of circulating FGF23 protein in *Cx43*^*KI/KI*^ mice. Lack of correlation between RNA synthesis and protein levels has been previously reported with possible explanations of post-transcriptional regulation, measurement noise, and lack of temporal synchronization.^68,69^ In disease models, it has been shown, especially in non-proliferating tissues, that protein levels change less than mRNA levels.^70^ The changes in transcription may provide for a partial buffer to ensure that proteins can respond more rapidly to a stimulus.^70^

In conclusion, our study presents a valid mouse model for investigating the molecular mechanisms of AR CMD. The R239Q mutation in Cx43 affects osteoblasts, osteoclasts, and osteocytes, collectively contributing to the AR CMD-like skeletal phenotype. Intracellular mislocalization and differential gene expression provides insights into possible mechanisms for observed bone abnormalities in *Cx43*^*KI/KI*^ mice. The unique skeletal features seen in the AR CMD *Cx43*^*KI/KI*^ mouse model suggest a yet unknown role of Cx43_R239Q_ in addition to a functional reduction of the Cx43_R239Q_ protein.

## Materials and methods

### Generation of the Cx43_R239Q_ knockin (KI) mouse model and study approval

To generate Cx43_R239Q_ KI mice (*Cx43*^*KI/KI*^), we used CRISPOR (http://crispor.tefor.net) to identify the *Cx43* target site, 5ʹ-GGGCGTTAAGGATCGCGTGAAGG with the protospacer adjacent motif (PAM) for CRISPR/Cas9 mediated gene editing. *Cx43* sgRNA and Cas9 protein were mixed at room temperature for 15 min to form the RNP complex prior to adding *Cx43* R239Q ssDNA donor (5ʹ-AT*T*G*AAGTAAGCATATTTTGGAGATCCGCAGTC TTTGGATGGGCTCAGTGGGCCGGTGGTGGCGTGGTAAGGATCGCTTCTTCCTTT CAC**CTG**ATCTTTAACGCCCTTGAAGAAGACATAGAAGAGCTCAATGATATTCAG AGCGAGAGAC*A*C*C-3ʹ) for pronuclear microinjection into C57BL/6 J one-cell embryos. The concentration of sgRNA, Cas9 and ssDNA donor was 50 ng, 200 ng and 50 ng per μL, respectively (all reagents from Integrated DNA Technologies, Coralville, IA). Injected embryos were transferred into pseudo-pregnant females and potential founders were screened by PCR using primers (CxF1: 5ʹ-GCTTCCTCTCACGTCCCACGGAG-3ʹ and CxR239QR: 5ʹ- GGATCGCTTCTTCCTTTCACCT-3ʹ) to amplify a fragment of 145 bp specific to the KI allele. Genotype of PCR-positive founders were further confirmed by PCR using primers (CxF1 and CxR1: 5ʹ-GCTTGTTGTAATTGCGGCAGGAGG-3ʹ) followed by sequencing of the PCR product (Fig. S8a, S8b). The R239Q mutation (CGC → CAG, pink bolded) and two silent mutations (blue bolded) were introduced to eliminate re-cleavage of the KI allele (Fig. S8a). A positive founder was bred with wildtype C57BL/6 J mice (Stock number: 000664, The Jackson Laboratory). Mice were housed in an AAALAC-accredited facility under veterinary supervision. All experiments involving animals were in accordance with animal protocol AP-200644-0025 approved by the Animal Care Committee at the University of Connecticut Health (UConn Health).

### Skeletal analysis

Skulls, mandibles, and femurs of male and female *Cx43*^*+/+*^ and *Cx43*^*KI/KI*^ mice at ages of 3-12 months (*n* ≥ 5 for each group) were radiographed with a Kubtec Radiography System (KUB Technologies Inc., Stratford, CT) and analyzed by μCT (mCT20; ScanCo Medical AG, Bassersdorf, Switzerland) in the MicroCT facility at UConn Health as previously described.^71^ Mandibular data were collected by measuring vertical sections covering 1st & 2nd molars. Femoral trabecular data were taken at the distal growth plate over a distance of 960 μm. Cortical bone parameters were collected from 50 cross-sectional slices of 12 μm in the mid-diaphysis.

For the analysis of bone histomorphometry, we injected male and female *Cx43*^+/+^ (*n* = 8) and *Cx43*^*KI/KI*^ (*n* = 8) mice intraperitoneally with calcein (10 mg/kg body weight) and alizarin complexone (AC, 30 mg/kg body weight) at an interval of 7 days. Two days after the second injection, mice were sacrificed at 13 weeks of age and bones subjected to histomorphometry as previously described.^71^ Femurs were fixed in 10% formalin and frozen-embedded in OCT medium (Richard-Allan Scientific, San Diego, CA). Blocks were sectioned using a cryotome (CM3050S; Leica, Wetzlar, Germany). Three sections (7 μm thickness) revealing the central vein were collected using adhesive tape (Cryofilm type IIC; Section-Lab Co, Ltd., Yokohama, Japan).^71^ For dynamic histomorphometry, fluorescent images showing the mineralization status by calcein (green) and AC (red) were taken using a microscope slide scanner (Mirax Midi automated image acquisition system; Carl Zeiss, Jena, Germany). Parameters of bone forming activity were measured in the Computer Science Department at UConn.^71,72^

After scanning for mineralization imaging, the same sections were subjected to cellular staining. To detect OBs, ALP was stained by the fluorescent substrate fast red.^73^ AP-positive cells located on calcein- or AC-labeled bone surfaces were considered active osteoblastic cells whereas AP-positive cells on non-labeled bone surfaces were considered inactive bone lining cells.^71,72^ For OCs, tartrate resistant acid phosphatase (TRAP) staining was performed using a fluorescent substrate (ELF-97; Thermo Fisher Scientific, Waltham, MA). Bone surfaces that had both, mineralization labeling and TRAP signals, were considered remodeling surfaces whereas surfaces with TRAP-positive cells but no mineralization labeling were considered resorbing surfaces. The TRAP and AP signals were captured using filters optimized for tetracycline and tetramethyl rhodamine iso-thiocyanate (TRITC), respectively.

### Biochemical analysis

Fasting sera were collected from the submandibular vein using animal lancets (Medipoint, Long Island, NY) in microtainer tubes (Becton Dickinson, Franklin Lakes, NJ) from 3-month- and 8-month-old *Cx43*^*+/+*^ and *Cx43*^*KI/KI*^ mice. Total serum calcium and phosphate concentrations were determined using a calcium reagent kit and a phosphorus reagent set (Eagle Diagnostics, Cedar Hill, Tx). Concentrations of mouse CTX (RatLaps CTX-1 ELISA kit; Immunodiagnostic Systems, East Boldon, United Kingdom), P1NP (Rat/mouse P1NP ELISA kit; Immunodiagnostic Systems, Mountain Lakes, NJ), FGF-23 (intact), and FGF-23 (C-terminal) (Mouse/Rat FGF-23 (intact) ELISA, Mouse/Rat FGF-23 (C-Term) ELISA; Quidel, SanDiego, CA) were determined according to manufacturer instructions.

### Mouse osteoblast cultures

To study OBs, we used mouse calvarial osteoblast (mCOB) cultures and bone marrow stromal cell (BMSC) cultures. For mCOB cultures, calvariae from postnatal day 4–7 mice were digested with 0.05% trypsin (Thermo Fisher Scientific) and 0.15% collagenase (Type II; Sigma Aldrich, St. Louis, MO) at 37 °C. Cells from digests 3–5 were collected and plated at a density of 10 000/cm^2^ in DMEM (Thermo Fisher Scientific) until confluent. Cells were maintained in osteoblast differentiation medium (α-ΜΕΜ; Thermo Fisher Scientific) containing 10% fetal bovine serum (FBS; Cytiva Life Sciences, Marlborough, MA), 100 IU/mL penicillin, 100 μg/mL streptomycin (Thermo Fisher Scientific), 50 μg/mL ascorbic acid and 4 mmol/L β-glycerophosphate (Sigma Aldrich). Medium was changed every 2–3 days. For BMSCs, bone marrow was flushed out from the shafts of femurs, tibiae and humeri of 7- to 9-week-old mice.^74^ Cells were cultured at a density of 2 × 10^6^ cells/well in 12-well culture plates in α-MEM containing 10% FBS, 100 IU/mL penicillin, and 100 μg/mL streptomycin. At day 3, half of the medium was changed. On day 7, cells were switched to 100% osteoblast differentiation medium containing 50 μg/mL ascorbic acid and 8 mmol/L β-glycerophosphate (β-GP). Medium was changed every 2–3 days.

### Mouse osteoclast cultures

Bone marrow-derived macrophage (BMM) cultures were obtained from bone marrow flushed out from femora and tibiae of 7- to 9-week-old mice and cultured for 18–24 h in α-MEM containing 10% FBS, 100 IU/mL penicillin, 100 μg/mL streptomycin. Non-adherent cells were collected and purified by Ficoll separation (Lymphoprep; STEMMCELL Technologies, Vancouver, Canada). Cells were cultured in α-MEM medium with 10% FBS, 100 IU/mL penicillin, 100 μg/mL streptomycin, and supplemented with M-CSF (30 ng/mL; Peprotech, Cranbury NJ) for 2 days to enrich OC progenitors followed by M-CSF and RANKL (30 ng/mL; Peprotech) treatment for 4-5 days and 9-10 days to obtain mature resting OCs (on culture plates) and actively resorbing OCs (on bone chips), respectively.

### In vitro osteoblast and osteoclast assays

For OB cultures, we determined matrix expression by ALP staining and mineral nodule formation by von Kossa staining. ALP staining was performed using a commercially available alkaline phosphatase kit (Sigma Aldrich) according to manufacturer instructions. Mineral deposition was stained with 5% silver nitrate (Sigma Aldrich).

We analyzed OC formation by TRAP staining with a commercially available kit (Sigma Aldrich).^75^ TRAP-positive stained cells ≥3 nuclei were counted as OCs. Actin rings of OCs were examined by rhodamine-phalloidin staining (1:40 dilution in PBS, Thermo Fisher Scientific). Nuclei were stained with Hoechst 33342 dye (trihydrochloride trihydrate; Molecular Probe, Thermo Fisher Scientific). Images were taken by a Z1 observer microscope (Carl Zeiss). For bone resorption pit assays, BMMs plated on bone chips were terminated at day 10 and bone chips were imaged using a tabletop scanning electron microscope (TM-1000, Hitachi, Tokyo, Japan). Quantitative data were obtained by Image J (National institutes of Health, NIH). Experiments were performed in triplicate.

### Quantitative real-time PCR (qPCR)

Total RNA from mCOBs, BMM cultures, and long bones (shafts of femurs and tibia without bone marrow) was isolated using TRIzol (Thermo Fisher Scientific) followed by Direct-zol RNA extraction (Zymo Research, Irvine, CA) according to manufacturer instructions. RNA was treated with DNase I and cDNA was synthesized by Superscript II reverse transcriptase (Invitrogen, Carlsbad, CA). qPCR was performed as previously described.^75^ Relative quantification of gene expression was determined by the 2^-ΔΔCt^ method. Data were normalized to mouse 18S gene expression. Primer sequences are listed in Table S2.

### Protein preparation, SDS-PAGE, and Western blotting analysis

Dissected tissues were immediately frozen by liquid nitrogen and crushed to powder. Two ends of femurs and tibiae were cut off followed by brief centrifugation to remove bone marrow. Cell lysate collected from mCOBs, BMMs, and osteocyte-like cells were subjected to immunoblots as described previously.^57^ Protein was extracted by lysis buffer (1% Triton X-100, 50 mmol/L Tris (pH 7.8), 150 mmol/L NaCl, 0.1% SDS, 10% glycerol, 0.5% deoxycholic acid) with protease and phosphatase inhibitor cocktails (Thermo Fisher Scientific). Protein concentrations were measured by BCA protein assay kit (Thermo Fisher Scientific). Equal amounts of protein were run in 10% SDS-PAGE gels. Samples were transferred to PVDF membranes (BioRad, Hercules, CA) using a wet transfer apparatus. Cx43 antibody (3512) was purchased from Cell Signaling Technology, Danvers, MA. GAPDH (Santa Cruz Biotechnology, Dallas, TX) served as internal control. Bands were detected using enhanced chemiluminescent detection reagent (Azure Biosystems, Dublin, CA) and visualized by an Azure c600 imaging system (Azure Biosystems). Densitometric analyses of immunoblots were performed by Image J.

### Cx43 Immunofluorescence of ovary cryosections, BMMs, and osteocyte-like cultures

Ovaries from *Cx43*^*+/+*^and *Cx43*^*KI/KI*^ mice were fixed in 4% paraformaldehyde (PFA, Electron Microscopy Sciences, Hatfield, PA) at 4 °C for 48 h, rinsed with 0.12 mol/L phosphate buffer, and cryo-protected with 30% sucrose at 4 °C overnight. 10 μm frozen sections were cut and collected on Superfrost plus slides (Thermo Fisher Scientific). Sections were rinsed with 1x PBS, blocked with 5% normal goat serum in 1% BSA in PBS, then incubated in anti-Cx43 antibody (C6219; Millipore Sigma, Burlington, MA) diluted 1:300 in blocking buffer for two hours at room temperature. After rinsing with PBS, sections were incubated in Alexa 488- goat anti-rabbit (Invitrogen) diluted 1:500 for 30 min at room temperature. Sections were imaged on a Zeiss Pascal confocal microscope with the same conditions for both genotypes.

Osteocyte-like cultures were prepared by a series of enzymatic digestions following a published method.^76^ Cells from fractions 6 to 9 and remaining bone chips were cultured on collagen-coated plates for 7 days. Osteocyte-like cells and multinucleated BMMs were fixed in 4% PFA for 10 min at room temperature. Cells were permeabilized with 0.2% Tween-20 for 3 min, washed with PBS, and blocked in 5% normal goat serum for 60 min at RT. Cells were incubated in anti-Cx43 antibody (C6219; Millipore Sigma) diluted 1:500 overnight. After rinsing with PBS three times, cells were incubated with goat anti-rabbit secondary antibody (1:400) for 1 h at RT in dark. Images were taken by a Z1 observer microscope (Carl Zeiss).

### Hemichannel and gap junction activity of Cx43

To assess the hemichannel activity, we cultured *Cx43*^*+/+*^ and *Cx43*^*KI/KI*^ skin fibroblasts and osteocyte-like cells at the density of 5000 and 10 000 cells per well in 96-well plates, respectively. The next day, cells were washed with PBS three times and cultured with or without 100 μmol/L Lucifer Yellow (LY) dye in DMEM containing 3 mmol/L EGTA for 30 min. Cells were then washed and fixed in 4% PFA for 5 min prior to measuring dye uptake with an excitation peak at 428 nm and an emission peak at 536 nm in a microplate reader (Tecan Life Sciences, Maennedorf, Switzerland). The fluorescence reading was normalized to nuclei staining with Hoechst 33342 (excitation peak at 352 nm and emission peak at 454 nm).

To evaluate the gap junction activity, donor and recipient *Cx43*^*+/+*^ and *Cx43*^*KI/KI*^ skin fibroblasts were seeded at a density of 12 000/cm^2^ (day 0). The following day (day 1), recipient cells were labeled with 2.4 μmol/L DilC18 dye (Thermo Fisher Scientific) for 2 h. On day 2, donor cells were trypsinized and stained with 5 μmol/L Calcein Violet AM (Thermo Fisher Scientific) for 20 min. Donor cells were then seeded on top of the recipient cells (Ratio of donor: recipient cells = 4:1) for 2 and 5 h. At the end points, cells were harvested and analyzed by flow cytometry using a Becton-Dickinson LSRII analyzer (Becton, Dickinson and Company, Franklin Lakes, NJ).

### Statistical analysis

Statistical analysis was performed by Student’s *t* test or two-way ANOVA followed by Tukey’s multiple comparison test, using Prism 5 (GraphPad Software, La Jolla, CA). Results of 2-way ANOVA analyses are summarized in Table S1.

## Supplementary information

supplemental file
