# Supplementary material for: Skeletal abnormalities caused by a Connexin43R239Q mutation in a mouse model for autosomal recessive craniometaphyseal dysplasia
Source: Bone Res. 2025 Jan 23;13:14. doi: 10.1038/s41413-024-00383-z (PMC11757998; doi:10.1038/s41413-024-00383-z)
Supplement: Supplementary file 1 — supplemental file [file 41413_2024_383_MOESM1_ESM.docx]

Skeletal abnormalities caused by a Connexin43_R239Q_ mutation in a mouse model for autosomal recessive craniometaphyseal dysplasia

Yasuyuki Fujii^1^, Iichiro Okabe^1^, Ayano Hatori^1^, Shyam Kishor Sah^1^, Jitendra Kanaujiya^2^, Melanie Fisher^2^, Rachael Norris^2^, Mark Terasaki^2^, Ernst J. Reichenberger^3^, and I-Ping Chen^1,3^*

^1^Department of Endodontology, School of Dental Medicine, University of Connecticut Health, Farmington, CT, United States

^2^Department of Cell Biology, University of Connecticut Health, Farmington, CT, United States

^3^Center for Regenerative Medicine and Skeletal Development, School of Dental Medicine, University of Connecticut Health, Farmington, CT, United States

Emails: yfujii@tokyo-med.ac.jp; [ichiandscratch@gmail.com](mailto:ichiandscratch@gmail.com); [hatori@uchc.edu](mailto:hatori@uchc.edu); [azzumax@gmail.com](mailto:azzumax@gmail.com); jitukanaujiya@yahoo.com; [fisher@uchc.edu](mailto:fisher@uchc.edu); [norris@uchc.edu](mailto:norris@uchc.edu); terasaki@uchc.edu; [reichenberger@uchc.edu](mailto:reichenberger@uchc.edu); [ipchen@uchc.edu](mailto:ipchen@uchc.edu)

Running title: A mouse model for recessive CMD

*Corresponding Author:

I-Ping Chen, DDS, PhD

(Professor)

University of Connecticut Health

Department of Endodontology

263 Farmington Avenue

Farmington, CT 06030-3705

Tel:    860-679-1030

Fax:   860-679-2910

email:  ipchen@uchc.edu


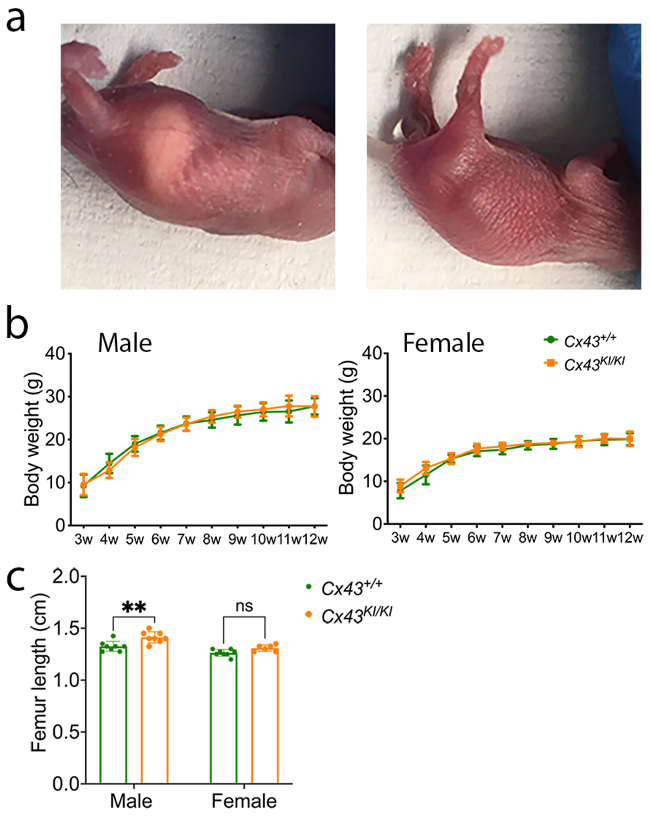


**Fig. S1**: **a**) Littermates (2 days old) with (left panel) and without (right panel) milk spot. **b**) Comparable body weight measured between ages of 3-12 weeks (w) in *Cx43^+/+^* and *Cx43^KI/KI^* male (left panel) and female (right panel) mice. **c**) Comparison of femur length between male and female *Cx43^+/+^* and *Cx43^KI/KI^* mice by two-way ANOVA analysis followed by Tukey’s multiple comparison test. ** *p*<0.001. ns: no statistically significant differences.


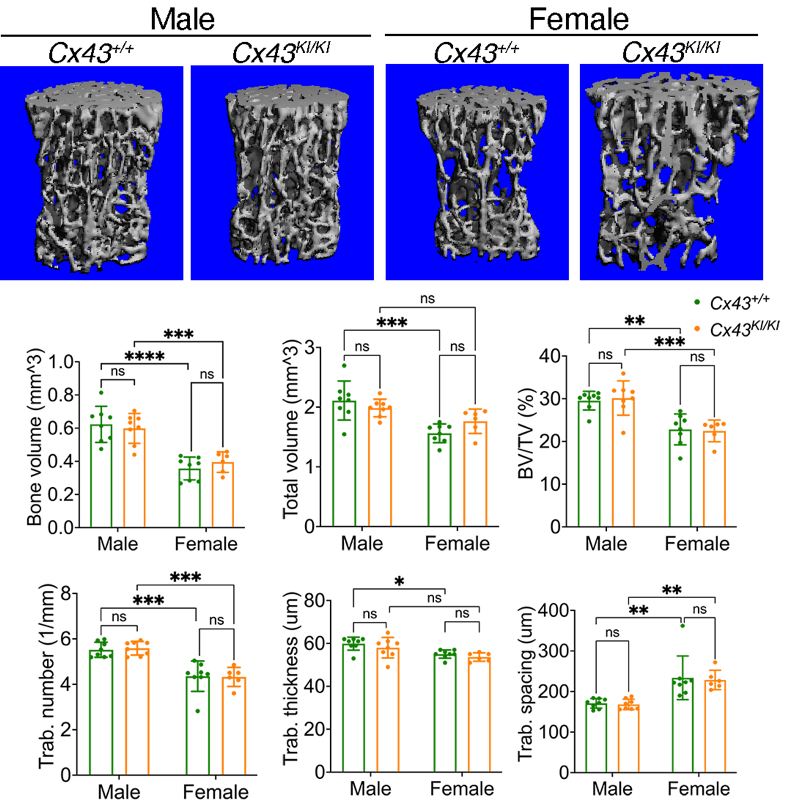


**Fig. S2**: μCT analysis of vertebrae. There are no significant differences in BV, TV, BVF (BV/TV), trabecular number, trabecular spacing and trabecular thickness between *Cx43^+/+^* and *Cx43^KI/KI^* mice. Statistics were performed by two-way ANOVA followed by Tukey’s post-hoc test (* *p*<0.05, ** *p*<0.01, *** and **** *p*<0.001).


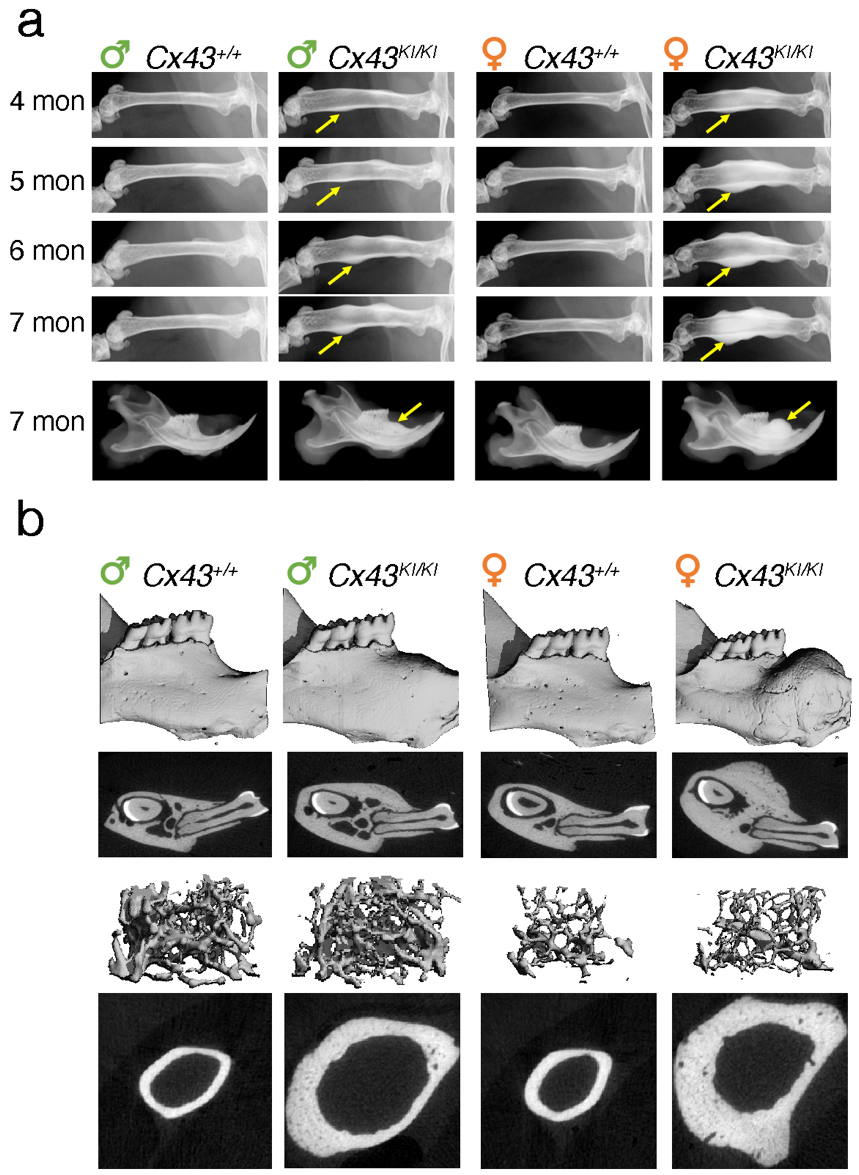


**Fig. S3**: Skeletal analysis of male and female *Cx43^+/+^* and *Cx43^KI/KI^* mice. **a**) Representative radiographs showing progressive changes of femoral phenotype from 4- to 7-month-old mice. Mandibular radiographs of 7-month-old mice. Yellow arrows indicated overgrowth of bone nodules of femurs and mandibles in *Cx43^KI/KI^* mice. **b**) Representative μCT images of mandibles and femoral trabeculation and cortical bones of 7-month-old male and female *Cx43^+/+^* and *Cx43^KI/KI^* mice.


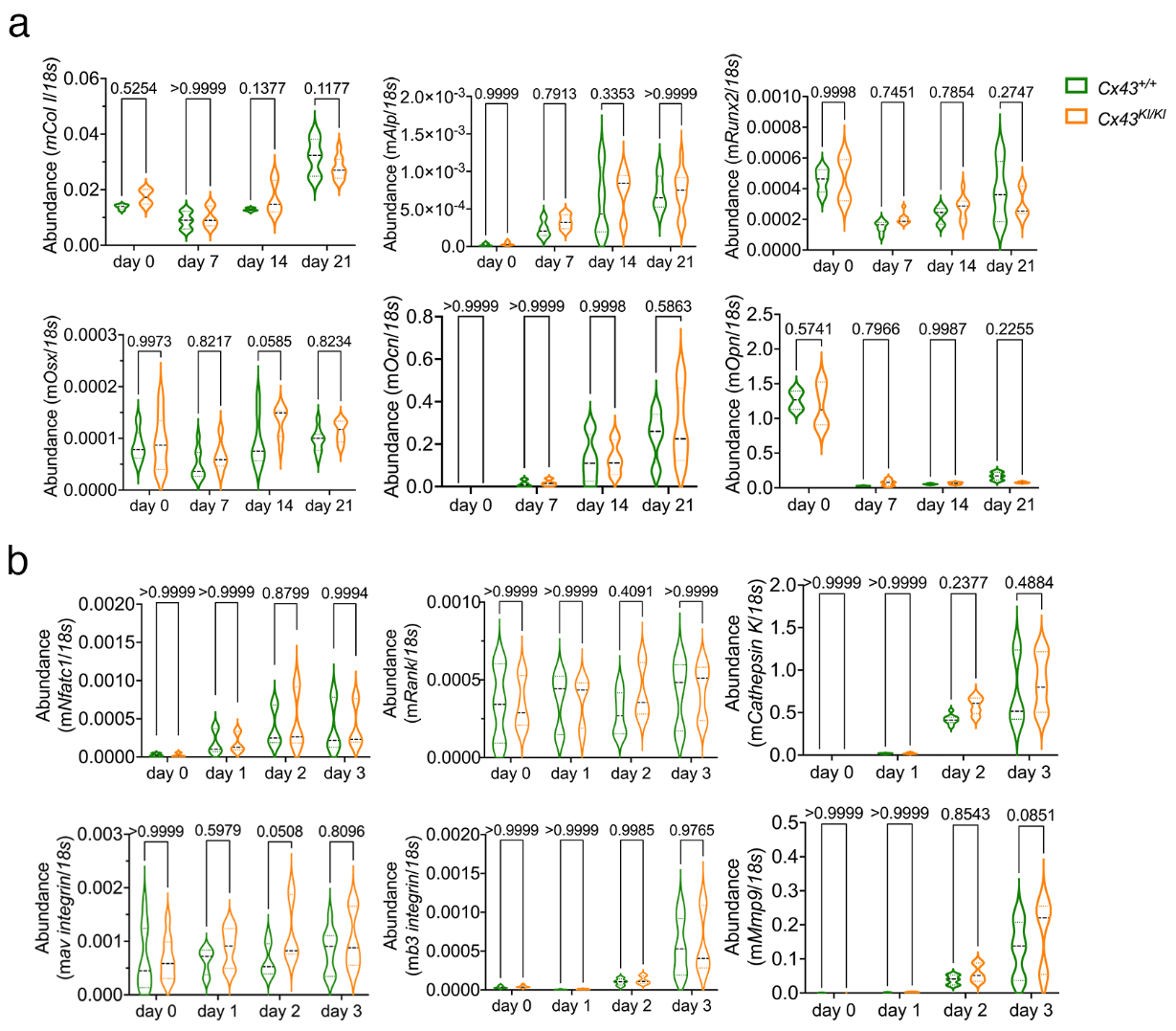


**Fig. S4**: Gene expression in mCOB cultures and BMM cultures by qPCR. **a**) Expression levels of *ColI*, *Alp*, *Runx2*, *Osx*, *Ocn*, and *Opn* in mCOBs differentiated in osteogenic medium for days 0, 7, 14, and 21. **b**) Expression levels of *Nfatc1*, *Rank*, *Cathepsin K*, *αv integrin*, *β3 integrin*, and *Mmp9* in BMMs cultured in MCSF and RANKL for days 0, 1, 2, and 3.


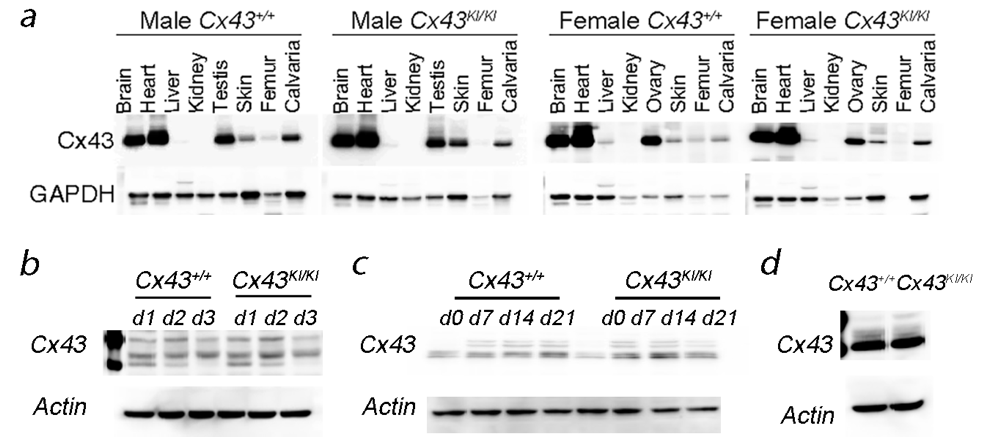


**Fig. S5**: Expression of *Cx43^KI/KI^* protein in **a**) multiple tissues; **b**) BMM cultures treated with MCSF for 2 days followed by MCSF and RANKL for 1, 2, and 3 days (d1, d2, and d3); **c**) mCOBs cultured in differentiating medium for 0, 7, 14 and 21 days (d0, d7, d14, and d21); **d**) osteocyte-like cultures from *Cx43^+/+^* and *Cx43^KI/KI^* mice by immunoblotting. GAPDH and actin serve as loading controls.


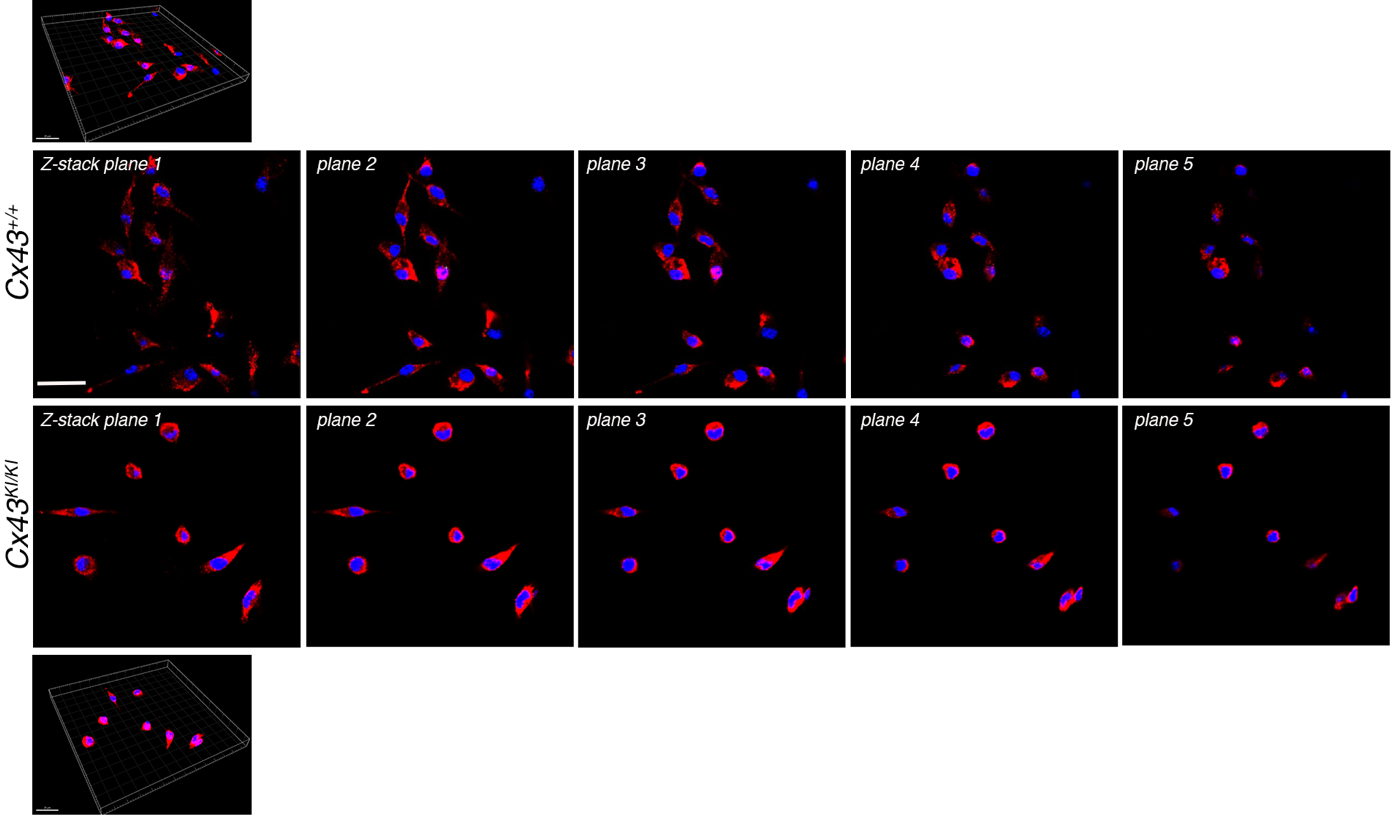


**Fig. S6**: Z-stack confocal images showing Cx43 immunocytochemistry of *Cx43^+/+^* and *Cx43^KI/KI^* osteocyte-like cells. Red: Cx43; blue: Hoechst 33342 nuclei staining. Scale bar = 20 μm.


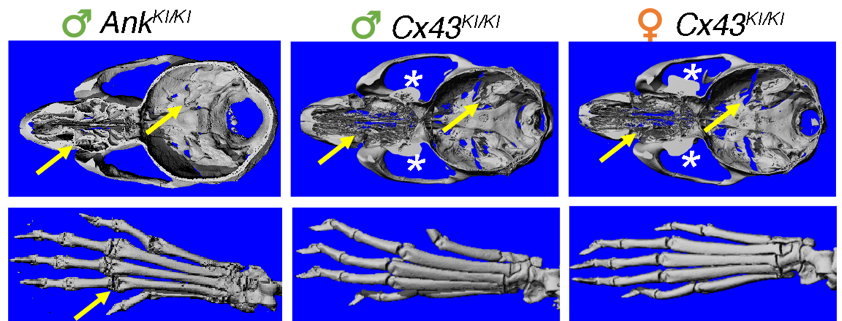


**Fig. S7**: Comparison of cranial base and feet from 3-month-old *Ank^KI/KI^* mice and 1-year-old male and female *Cx43^KI/KI^* mice. Yellow arrows indicate more severe obstruction of nasal cavity, narrowing cranial foramina, and excessive mineral deposition of joints in *Ank^KI/KI^* mice. White asterisks indicate overgrowth of bone in *Cx43^KI/KI^* mice but not *Ank^KI/KI^* mice.


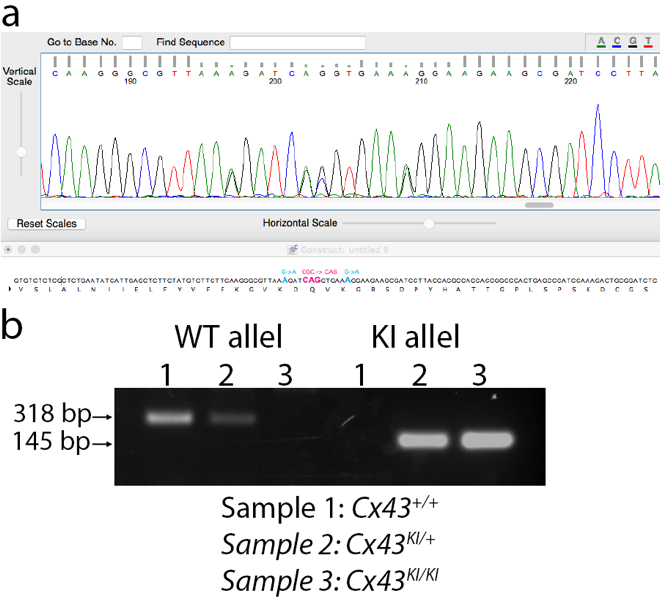


**Fig. S8**: **a**) Sequence of F1 heterozygous Cx43 KI (*Cx43^KI/+^*) mice. Pink letters showing the introduction of the R239Q mutation (CGC 🡪 CAG). **b**) A representative gel image of genotyping results for *Cx43^+/+^*, *Cx43^KI/+^*, and *Cx43^KI/KI^* mice.

**Table S1**: Results of 2-way ANOVA analyses presented in the manuscript. Data presented are *p*-values of row factor, column factor and interaction

| *Parameters in Fig. 1b* | *Sex (row factor)* | *Genotype (column factor)* | *Interaction* |
| --- | --- | --- | --- |
| Bone volume | 0.7999 | **<0.0001** | 0.3308 |
| Total volume | 0.4289 | **<0.0001** | 0.4860 |
| BV/TV | **0.0007** | **<0.0001** | 0.1483 |
| *Parameters in Fig. 1c* | *Sex (row factor)* | *Genotype (column factor)* | *Interaction* |
| Bone volume | **<0.0001** | 0.6448 | 0.7366 |
| Total volume | **<0.0001** | **<0.0001** | 0.7986 |
| BV/TV | **<0.0001** | 0.0811 | 0.6608 |
| Trab. Number | **<0.0001** | 0.2392 | 0.8753 |
| Trab. Spacing | 0.2464 | **<0.0001** | 0.7825 |
| Trab. Thickness | 0.7905 | **<0.0001** | 0.5329 |
| Sub-periosteal area | **<0.0001** | **<0.0001** | **0.0008** |
| Sub-endosteal area | **<0.0001** | **<0.0001** | 0.8489 |
| Cortical porosity | **0.0018** | **<0.0001** | **<0.0001** |
| *Parameters in Fig. 2a* | *Sex (row factor)* | *Genotype (column factor)* | *Interaction* |
| MS/BS  (Periosteum) | 0.0899 | **<0.0001** | 0.0590 |
| MAR  (Periosteum) | **<0.0001** | **<0.0001** | **<0.0001** |
| BFR  (Periosteum) | **<0.0001** | **<0.0001** | **<0.0001** |
| MS/BS  (Endosteum) | **<0.0001** | **<0.0001** | 0.6917 |
| MAR  (Endosteum) | **<0.0001** | **<0.0001** | 0.6780 |
| BFR  (Endosteum) | **<0.0001** | **<0.0001** | **0.0356** |
| *Parameters in Fig. 2b* | *Sex (row factor)* | *Genotype (column factor)* | *Interaction* |
| TRAP+ area/bone perimeter  (Periosteum) | 0.9335 | **<0.0001** | 0.9335 |
| TRAP+ area/bone perimeter  (Endosteum) | **<0.0001** | **<0.0001** | **<0.0001** |
| *Parameters in Fig. 3a* | *Sex (row factor)* | *Genotype (column factor)* | *Interaction* |
| P1NP (3-month-old) | 0.1783 | **0.0084** | 0.9925 |
| CTX (3-month-old) | 0.6383 | 0.4817 | 0.5962 |
| P1NP (8-month-old) | 0.5968 | **0.0022** | 0.5763 |
| CTX (8-month-old) | 0.5291 | 0.9770 | 0.5708 |
| *Parameters in Fig. 3b* | *Sex (row factor)* | *Genotype (column factor)* | *Interaction* |
| Calcium (3-month-old) | **<0.0001** | 0.2832 | 0.8185 |
| Pi (3-month-old) | **<0.0001** | **0.0251** | 0.5703 |
| Calcium (8-month-old) | 0.3224 | 0.3960 | 0.3972 |
| Pi (8-month-old) | 0.6457 | 0.1027 | 0.5173 |
| *Parameters in Fig. 3c* | *Sex (row factor)* | *Genotype (column factor)* | *Interaction* |
| FGF23 intact | **0.036** | 0.3105 | 0.3218 |
| FGF23 C-terminal | 0.3413 | 0.3433 | 0.0951 |
| *Parameters in Fig. 5b* | *Day (row factor)* | *Genotype (column factor)* | *Interaction* |
| Mineral nodules/total area | **<0.0001** | 0.5613 | 0.4389 |
| *Parameters in Fig. 5c* | *Day (row factor)* | *Genotype (column factor)* | *Interaction* |
| Abundance (*Rankl/18S*) | **<0.0001** | **0.0233** | **0.05** |
| Abundance (*Opg/18S*) | **<0.0001** | **<0.0001** | **<0.0001** |
| Ratio (*Rankl/Opg*) | **<0.0001** | 0.8452 | 0.1814 |
| *Parameters in Fig. 5d* | *Day (row factor)* | *Genotype (column factor)* | *Interaction* |
| ALP | **<0.0001** | 0.5554 | 0.9577 |
| Nodules/total well area | **<0.0001** | 0.5613 | 0.4389 |
| *Parameters in Fig. 6a* | *Sex (row factor)* | *Genotype (column factor)* | *Interaction* |
| TRAP^+^ multinucleated cells/well | 0.1767 | 0.3972 | 0.0664 |
| *Parameters in Fig. 7c – 7f* | *Sex (row factor)* | *Genotype (column factor)* | *Interaction* |
| Abundance (*Fgf23/18S*) | **<0.0001** | **0.0135** | 0.0019 |
| Abundance (*Phex/18S*) | **0.0042** | 0.1532 | 0.0098 |
| Abundance (*Dmp1/18S*) | **0.0004** | 0.9031 | 0.0859 |
| Abundance (*Tnfα/18S*) | 0.1698 | **<0.0001** | 0.0358 |
| Abundance (*IL-1β/18S*) | **0.0043** | **0.0215** | **0.01** |
| Abundance (*Esr1/18S*) | **0.0009** | **<0.0001** | **0.0005** |
| Abundance (*Esr2/18S*) | **0.0903** | **<0.0001** | **0.0122** |
| Abundance (*Sost/18S*) | **0.0011** | **<0.0001** | **0.0078** |
| Abundance (*Rankl/18S*) | 0.7505 | **<0.0001** | 0.1549 |
| Abundance (*Opg/18S*) | 0.4381 | **0.0347** | **0.0127** |
| Ratio (*Rankl/Opg*) | 0.0396 | **<0.0001** | 0.1219 |
| *Parameters in Fig. 8d* | *LY (row factor)* | *Genotype (column factor)* | *Interaction* |
| Hemichannel activity (lucifer yellow/DAPI) | **<0.0001** | **0.0004** | 0.067 |
| *Parameters in Table 1* | *Sex (row factor)* | *Genotype (column factor)* | *Interaction* |
| MAR | 0.3485 | 0.7288 | 0.0826 |
| BFR | 0.0593 | 0.8056 | 0.1342 |
| AP/BS | **0.0141** | 0.4316 | 0.1869 |
| AP_L/BS | 0.9789 | 0.7425 | 0.2668 |
| AP_NL/BS | **0.0002** | 0.2364 | 0.2997 |
| TRAP/BS | **<0.0001** | 0.79 | **0.0104** |
| TRAP_L/BS | **<0.0001** | 0.5395 | 0.0894 |
| TRAP_NL/BS | **<0.0001** | 0.4777 | 0.1655 |
| AP_TRAP_R/BS | **<0.0001** | 0.6985 | **0.0318** |
| *Parameters in Fig. S1c* | *Sex (row factor)* | *Genotype (column factor)* | *Interaction* |
| Femur length | **<0.0001** | **0.0003** | 0.2006 |
| *Parameters in Fig. S2* | *Sex (row factor)* | *Genotype (column factor)* | *Interaction* |
| Bone volume | **<0.0001** | 0.8240 | 0.3249 |
| Total volume | **<0.0001** | 0.6444 | 0.0565 |
| BV/TV | **<0.0001** | 0.912 | 0.689 |
| Trab. Number | **<0.0001** | 0.9112 | 0.7641 |
| Trab. Thickness | **<0.0001** | 0.1879 | 0.8212 |
| Trab. Spacing | **<0.0001** | 0.7343 | 0.9088 |
| *Parameters in Fig. S4* | *Day (row factor)* | *Genotype (column factor)* | *Interaction* |
| Abundance (*ColI/18S*) | **<0.0001** | 0.2712 | **0.0142** |
| Abundance (*Alp/18S*) | **<0.0001** | 0.1665 | 0.6532 |
| Abundance (*Runx2/18S*) | **<0.0001** | 0.8298 | 0.1592 |
| Abundance (*Osx/18S*) | **0.0003** | **0.0273** | 0.5124 |
| Abundance (*Ocn/18S*) | **<0.0001** | 0.2739 | 0.4993 |
| Abundance (*Opn/18S*) | **<0.0001** | 0.2841 | 0.1478 |
| Abundance (*Nfatc1/18S*) | **<0.0001** | 0.6359 | 0.9181 |
| Abundance (*Rank/18S*) | 0.5096 | 0.4069 | 0.6269 |
| Abundance (*Cathepsin K/18S*) | **<0.0001** | 0.0996 | 0.4305 |
| Abundance (*av integrin/18S*) | 0.2750 | **0.0183** | 0.3631 |
| Abundance (*b3 integrin/18S*) | **<0.0001** | 0.6642 | 0.9858 |
| Abundance (*Mmp9/18S*) | **<0.0001** | 0.1056 | 0.3177 |

**Table S2**: qPCR primer sequences

| Genes | Forward primer | Reverse primer |
| --- | --- | --- |
| *ColI* | 5’-TCTCCACTCTTCTAGTTCCT | 5’-TTGGGTCATTTCCACATGC |
| *Alp* | 5’-GCCCTCTCCAAGACATATA | 5’-CCATGATCACGTCGATATCC |
| *Runx2* | 5’-CCGCACGACAACCGCACCAT | 5’-CGCTCCGGCCCACAAATCTC |
| *Osx* | 5’-GGATGGCGTCCTCTCTGCTTGAG | 5’-GAGGAGTCCATTGGTGCTTGAGA |
| *Ocn* | 5’-AAGCAGGAGGGCAATAAGGT | 5’-AGCTGCTGTGACATCCATAC |
| *Opn* | 5’-TCACCATTCGGATGAGTCTG | 5’-ACTTGTGGCTCTGATGTTCC |
| *Nfatc1* | 5'-CTCGAAAGACAGCACTGGAGCAT | 5'-CGGCTGCCTTCCGTCTCATAG- |
| *Rank* | 5’′-TCTCAGATGTCTTTTCGTCCACAG | 5′-AGCCACTACTACCACAGAGATG |
| *Cathepsin K* | 5’-TGTATAACGCCACGGCAAA | 5’-GGTTCACATTATCACGGTCACA |
| *αv integrin* | 5’-CCTCAGAGAGGGAGATGTTCACAC | 5’-AACTGCCAAGATGATCACCCACAC |
| *β3 integrin* | 5’-GATGACATCGAGCAGGTGAAAGAG | 5’-CCGGTCATGAATGGTGATGAGTAG |
| *Mmp9* | 5’-TCCAGTACCAAGACAAAGCCTA | 5’-TTGCACTGCACGGTTGAA |
| *Rankl* | 5’-CAC CAT CAG CTG AAG ATA GT | 5’- CCA AGA TCT CTA ACA TGA CG |
| *Opg* | 5’- AGA GCA AAC CTT CCA GCT GC | 5’- CTG CTC TGT GGT GAG GTT CG |
| *Fgf23* | 5’-ACTTGTCGCAGAAGCATC | 5’-GTGGGCGAACAGTGTAGAA |
| *Phex* | 5’-GCATGATTAACCAGTATAGCAA | 5’-GGTCTATAGGAATTGCACCTTAC |
| *Dmp1* | 5’-GCGCGGATAAGGATGA | 5’-GTCCCCGTGGCTACTC |
| *Tnfa* | 5’-CGCTCTTCTGTCTACTGAAC | 5’-TGTCCCTTGAAGAGAACCTG |
| *IL-1b* | 5’-CAACCAACAAGTGATATTCTCCATG | 5’-GATCCACACTCTCCAGCTGCA |
| *Esr1* | 5’-TGCAATGACTATGCCTCTGG | 5’-TTCAACATTCTCCCTCCTCG |
| *Esr2* | 5’-GAACTGGTGCACATGATTGG | 5’-TCTTCGAAATCACCCAGACC |
| *Sost* | 5’-GCCGGACCTATACAGGACAA | 5’-CACGTAGCCCAACATCACAC |
